# Type I Interferon Upregulates Bak and Contributes to T Cell Loss during Human Immunodeficiency Virus (HIV) Infection

**DOI:** 10.1371/journal.ppat.1003658

**Published:** 2013-10-10

**Authors:** Joseph A. Fraietta, Yvonne M. Mueller, Guibin Yang, Alina C. Boesteanu, Donald T. Gracias, Duc H. Do, Jennifer L. Hope, Noshin Kathuria, Shannon E. McGettigan, Mark G. Lewis, Luis D. Giavedoni, Jeffrey M. Jacobson, Peter D. Katsikis

**Affiliations:** 1 Department of Microbiology and Immunology, Center for Immunology and Vaccine Science, Drexel University College of Medicine, Philadelphia, Pennsylvania, United States of America; 2 Translational Research Program, Abramson Cancer Center, University of Pennsylvania, Philadelphia, Pennsylvania, United States of America; 3 BIOQUAL, Inc., Rockville, Maryland, United States of America; 4 Departments of Virology and Immunology, Southwest National Primate Research Center, Texas Biomedical Research Institute, San Antonio, Texas, United States of America; 5 Department of Medicine, Division of Infectious Diseases and HIV Medicine, Drexel University College of Medicine, Philadelphia, Pennsylvania, United States of America; National Institute of Allergy and Infectious Diseases, National Institutes of Health, United States of America

## Abstract

The role of Type I interferon (IFN) during pathogenic HIV and SIV infections remains unclear, with conflicting observations suggesting protective versus immunopathological effects. We therefore examined the effect of IFNα/β on T cell death and viremia in HIV infection. *Ex vivo* analysis of eight pro- and anti-apoptotic molecules in chronic HIV-1 infection revealed that pro-apoptotic Bak was increased in CD4+ T cells and correlated directly with sensitivity to CD95/Fas-mediated apoptosis and inversely with CD4+ T cell counts. Apoptosis sensitivity and Bak expression were primarily increased in effector memory T cells. Knockdown of Bak by RNA interference inhibited CD95/Fas-induced death of T cells from HIV-1-infected individuals. In HIV-1-infected patients, IFNα-stimulated gene expression correlated positively with *ex vivo* T cell Bak levels, CD95/Fas-mediated apoptosis and viremia and negatively with CD4+ T cell counts. *In vitro* IFNα/β stimulation enhanced Bak expression, CD95/Fas expression and CD95/Fas-mediated apoptosis in healthy donor T cells and induced death of HIV-specific CD8+ T cells from HIV-1-infected patients. HIV-1 *in vitro* sensitized T cells to CD95/Fas-induced apoptosis and this was Toll-like receptor (TLR)7/9- and Type I IFN-dependent. This sensitization by HIV-1 was due to an indirect effect on T cells, as it occurred in peripheral blood mononuclear cell cultures but not purified CD4+ T cells. Finally, peak IFNα levels and viral loads correlated negatively during acute SIV infection suggesting a potential antiviral effect, but positively during chronic SIV infection indicating that either the virus drives IFNα production or IFNα may facilitate loss of viral control. The above findings indicate stage-specific opposing effects of Type I IFNs during HIV-1 infection and suggest a novel mechanism by which these cytokines contribute to T cell depletion, dysregulation of cellular immunity and disease progression.

## Author Summary

Type I interferons (IFNα/β) are innate immune mediators that are produced by cells in response to viral infections. Although the protective effects of IFNα/β are well-established, it is not clear whether these cytokines are beneficial or deleterious during HIV-1 infection. We report that HIV-1 infection renders T cells more prone to undergo programmed death and that this enhanced apoptosis susceptibility is associated with abnormal expression of pro- and anti-apoptotic molecules. Importantly, IFNα/β increases the expression level of the pro-apoptotic protein, Bak, a major gatekeeper of the mitochondrial apoptosis machinery. Exposure of healthy donor T cells to IFNα/β increased Bak expression and induced an apoptosis sensitivity that is similar to what is observed in HIV-1-infected patient T cells. Furthermore, elevated IFNα production and Bak expression correlated with heightened T cell apoptosis, low CD4+ T cell numbers and high viral loads in patients. In a primate model of HIV-1 infection, we observed that increased IFNα was associated with low peak viral loads in early infection, but high viremia and decreased CD4+ T cell counts during chronic infection. These studies identify a novel mechanism by which Type I IFN may induce immune dysfunction in HIV-1 infection.

## Introduction

Pathogenic HIV-1 infections are characterized by a generalized immune activation with concomitant CD4+ T cell depletion and the failure to effectively control viral replication. Increased apoptosis of uninfected T cells is observed in HIV-1-infected individuals and positively correlates with disease progression [1]. CD4+ T cells and CD8+ T cells from HIV-1-infected patients undergo elevated spontaneous apoptosis, activation-induced cell death (AICD), and CD95/Fas-mediated apoptosis [2], [3], [4], [5], [6], [7]. Although the exact mechanisms underlying this apoptosis are largely unknown, HIV-1-induced immune activation may contribute to the destruction of T cells and acquired immunodeficiency syndrome (AIDS) progression [8], implicating a role for cytokines in sensitizing T cells for apoptosis.

Type I IFNs (IFNα/β) are antiviral cytokines that are synthesized in response to the activation of molecular pattern recognition receptors by virus-specific molecules. Plasmacytoid dendritic cells (pDC) produce the majority of IFNα in response to Toll-like receptor (TLR)7 and TLR9 activation by HIV-1 [9]. Accordingly, IFNα is detected at elevated levels in the sera of HIV-1-infected and AIDS patients [10], [11], [12]. Type I IFN modulates innate and adaptive immune responses by decreasing viral replication [13] in a cell type-specific manner [14], regulating the differentiation of antigen-presenting cells [15], and promoting the proliferation or death of T cells [16]. Despite its well-characterized antiviral activity, the role of IFNα/β in HIV-1 infection remains controversial, with conflicting studies suggesting protective versus deleterious effects on host immunity. Although the level of immune activation in the early stages of HIV-1 infection is predictive of disease outcome [17], [18], the kinetics of Type I IFN production in relation to CD4+ T cell loss and viral control over time is largely unknown. IFNα production may be beneficial, as it has been demonstrated to directly suppress HIV-1 replication *in vitro* [19]. The administration of recombinant IFNα to HIV-1-infected individuals provides a modest therapeutic benefit [20], [21], but decreases CD4+ T cell counts during more advanced stages of HIV-1 disease [22]. In individuals infected with HIV-1 and rhesus macaques infected with simian immunodeficiency virus (SIV), plasma levels of IFNα remain elevated over time [10], as opposed to natural hosts of SIV [9], [23] and this may contribute to immunosuppression. In addition, persistent elevated IFNα production may impair thymopoiesis, bias T cell selection and induce generalized immune activation [24]. Furthermore, the expression of IFNα, Fas/FasL, and tumor necrosis factor–related apoptosis-inducing ligand (TRAIL)/death receptor (DR)5 is increased in the lymphoid tonsillar tissue of individuals with progressive HIV-1 disease, as compared to non-progressors who do not exhibit CD4+ T cell depletion [25]. It is therefore possible that Type I IFN exerts pathogenic effects during HIV-1 infection by facilitating the apoptotic death of T cells through mechanisms involving the tumor necrosis factor (TNF) receptor family.

To understand the role of Type I IFN in priming T cells for apoptosis, we investigated the effect of IFNα/β on pro- and anti-apoptotic molecules and the apoptosis sensitivity of T cells in HIV-1-infected patients. We found that *ex vivo* CD4+ T cells and CD8+ T cells, as well as HIV-specific CD8+ T cells from individuals with chronic HIV-1 infection are primed to undergo apoptosis through CD95/Fas but not TRAIL or TNFα signaling. Although Bak, Bax and Bim levels were significantly increased in T cells from HIV-1-infected patients, only Bak expression correlated inversely with CD4+ T cell counts and positively with CD95/Fas-mediated death. In addition, Bak knockdown by RNA interference prevented this apoptosis. Upon investigating what drives this apoptotic phenotype, we found that IFNα/β increased Bak expression and the sensitivity of healthy donor T cells, as well as HIV-specific CD8+ T cells to CD95/Fas-mediated death. In support of these findings, non-infectious HIV-1 sensitized healthy T cells to CD95/Fas-induced death and this apoptosis induction was mediated by TLR7/9 and Type I IFN. Further evidence for the pro-apoptotic role of Type I IFN and Bak in HIV-1 infection was revealed by our finding that *ex vivo* expression of the IFNα-induced genes [26] in PBMC from HIV-1-infected patients was increased and positively associated with Bak expression levels, CD95/Fas-mediated T cell apoptosis sensitivity and high viral loads, while it correlated inversely with CD4+ T cell counts. An inverse correlation between plasma IFNα levels and CD4+ T cell counts was also observed during chronic SIV infection. Finally, we show that although peak levels of plasma IFNα in SIV-infected rhesus macaques appear to contribute to viral control during the early stages of infection, persistent IFNα production was ultimately associated with loss of viral control and disease progression. Our findings indicate that although in acute infection Type I IFN may exert an important antiviral effect, during chronic HIV-1 infection, IFNα/β may contribute to the loss of CD4+ T cells and the failure of HIV-specific CD8+ T cells to control viral replication by upregulating Bak and sensitizing T cells to CD95/Fas-induced apoptosis. This study also suggests that Type I IFN has both beneficial and deleterious effects on the host immune response to pathogenic HIV-1 and SIV, depending on the stage of infection.

## Results

### *In vitro* T cells from HIV-1-infected individuals are sensitive to CD95/Fas-mediated apoptosis, but not to TRAIL or TNFα-induced death

**Figure 1:**
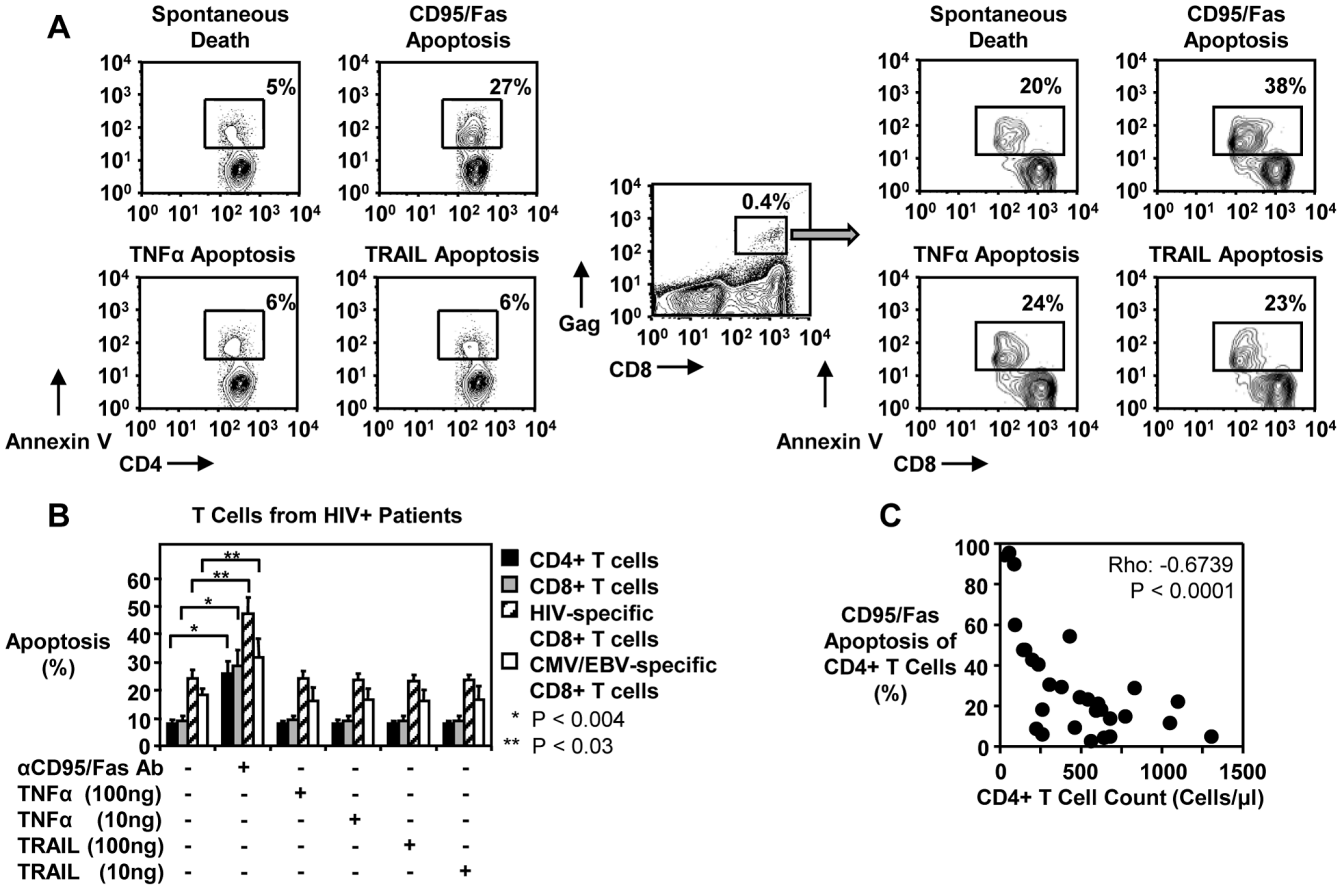
CD95/Fas-mediated, but not TRAIL- or TNFα-induced T cell apoptosis is increased in HIV-1-infected individuals and correlates inversely with CD4+ T cell counts. (A) Representative flow cytometry plots depicting apoptosis of CD4+ T cells and HIV-specific CD8+ T cells induced by anti-CD95/Fas antibody, SuperKiller TRAIL or TNFα treated PBMC from HIV-1+ individuals for 14 hours. (B) Pooled data showing percent apoptosis of CD4+ T cells (n = 8), total CD8+ T cells (n = 8), HIV-specific (n = 5) and CMV/EBV-specific (n = 5) CD8+ T cells after anti-CD95/Fas-, TRAIL-, and TNFα-treatment. Bars depict means ± standard errors. The P values were calculated using Student t test for paired samples. (C) Spearman's rho correlation shown between the frequency of CD95/Fas-mediated apoptosis of CD4+ T cells and absolute CD4+ T cell counts (n = 31).

We and others have previously reported that T cells from HIV-1-infected individuals are highly susceptible to CD95/Fas-induced apoptosis [5], [6], [7], [27], [28], [29]. However, the role of other TNF receptor family members in mediating increased T cell death in HIV-1-infected individuals is not well characterized. To address this, we examined the effects of TNFα and TRAIL on the survival of total CD4+ T cells, CD8+ T cells, as well as HIV-, CMV- and EBV-specific CD8+ T cells from HIV-1-infected patients. PBMC from HIV-1-infected subjects were incubated with media alone or with anti-CD95 antibody, TNFα or TRAIL for 14 hours and subsequently stained for apoptosis. While total CD4+ T cells, CD8+ T cells, HIV-specific and CMV/EBV-specific CD8+ T cells were susceptible to CD95/Fas-induced apoptosis (Figure 1A and B), neither TNFα nor TRAIL increased apoptosis above levels of spontaneous death (Figure 1A and B). In contrast, treatment of Jurkat T cells with anti-CD95/Fas antibody, SuperKiller TRAIL alone or TNFα with cyclohexamide induced similar apoptosis levels (data not shown). Furthermore, the enhanced susceptibility of CD4+ T cells to CD95/Fas-mediated death was strongly associated with CD4+ T cell loss in patients (Figure 1C). These *in vitro* data suggest that CD95/Fas is a predominant apoptotic pathway of the TNF receptor family in HIV-1 disease which contributes to the depletion of CD4+ T cells and impairs cytotoxic T cells.

### Bak upregulation in HIV-1 infection is associated with CD4+ T cell loss and controls CD95/Fas-mediated T cell apoptosis

**Figure 2:**
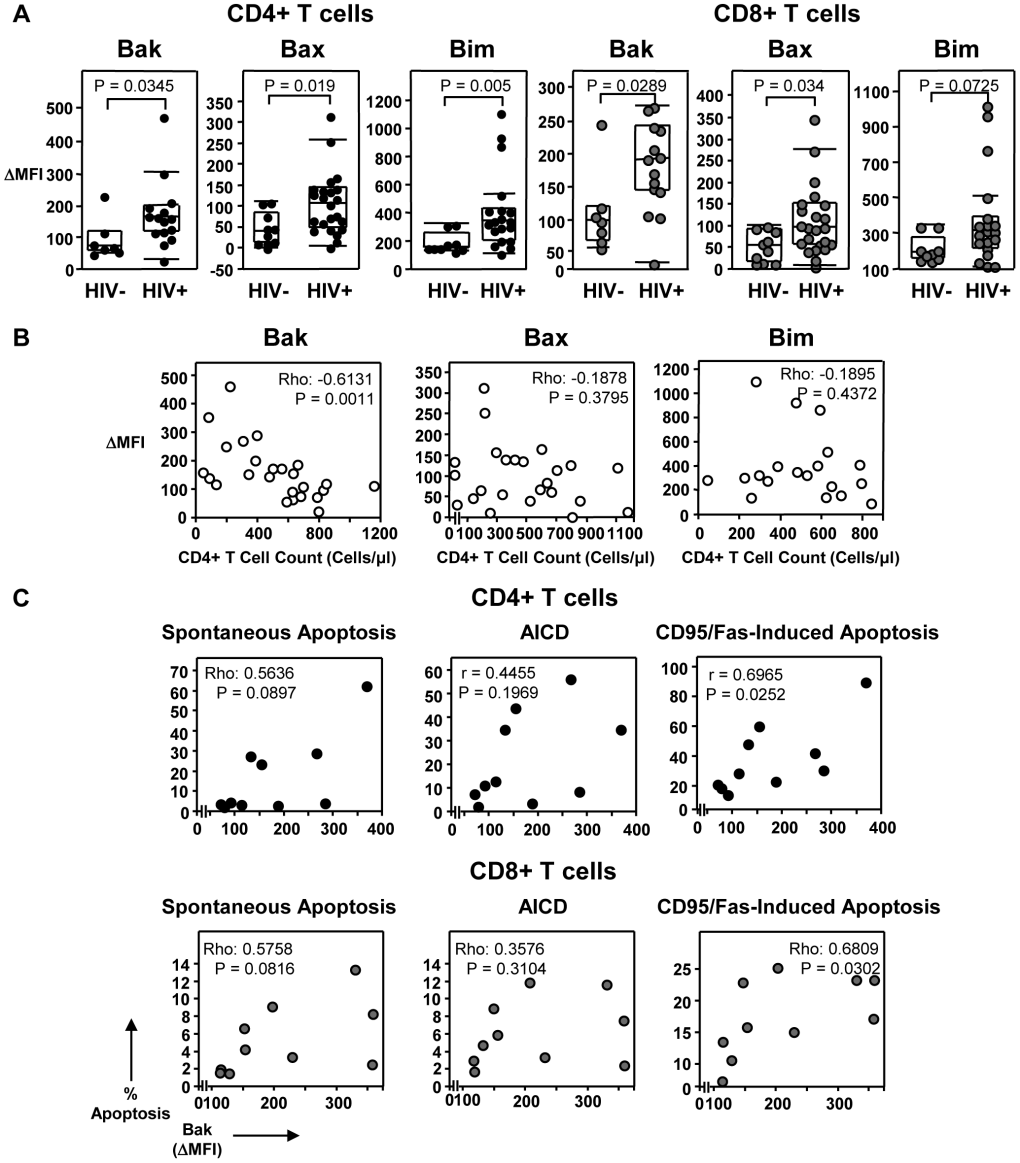
Bak expression is increased and correlates with CD4+ T cell counts and CD95-induced apoptosis in HIV-1-infected individuals. (A) Mean fluorescence intensity (MFI) of Bak, Bax and Bim expression *ex vivo* shown in CD4+ T cells and CD8+ T cells from HIV-1+ patients and healthy controls. Each dot depicts a donor, the lines indicate 10% and 90% and the boxes depict 25%, median and 75% quantiles. The P values were calculated by using the nonparametric Wilcoxon signed rank test for unpaired samples. (B) Spearman's rho correlation shown between *ex vivo* Bak (n = 25), Bax (n = 24) and Bim (n = 19) expression in CD4+ T cells and CD4+ T counts in HIV-1-infected individuals. (C) Spearman's rho or Pearson's r correlations shown between *ex vivo* Bak expression and the percentage of spontaneous apoptosis, AICD and CD95/Fas-mediate apoptosis in CD4+ and CD8+ T cells from HIV-1-infected individuals (n = 10).

We have previously demonstrated that Bcl-2 and Bcl-xL levels are significantly reduced in HIV-specific CD8+ T cells and correlate with high sensitivity to apoptosis [30]. Additionally, pro-apoptotic Bim and Bak are reported to be upregulated in CD4+ T cells from SIV-infected rhesus macaques [31]. However, the involvement of other Bcl-2-related proteins in T cell apoptosis during chronic HIV-1 infection is largely unknown. In order to address this, we examined *ex vivo* levels of pro-apoptotic members of the Bcl-2 family and other signaling molecules of the CD95/Fas pathway in T cells from HIV-1-infected subjects and uninfected donors. The expression of Bak, Bax and Bim was significantly increased in CD4+ T cells from HIV-1-infected patients, relative to healthy donors (Figure 2A; Figure S1; Table 1). Bak and Bax were also upregulated in CD8+ T cells from HIV-1-infected individuals, while Bim levels, although higher, did not significantly differ between HIV-1-infected and uninfected controls (Figure 2A, Table 1). A significant inverse correlation was observed between Bak expression and the absolute numbers of CD4+ T cells in HIV-1-infected patients (Figure 2B), whereas Bax and Bim were not associated with CD4+ T cell loss *in vivo* (Figure 2B). As previously reported in HIV-1-infected patients [28], [30], [32], Bcl-2 was decreased in CD8+ T cells (Table 1). *Ex vivo* levels of Bid and FADD in CD4+ T cells and CD8+ T cells did not differ between healthy donors and HIV-1-infected individuals (Table 1). These data suggest that while CD8+ T cells in chronic HIV-1 infection exhibit low levels of anti-apoptotic Bcl-2, pro-apoptotic Bak is increased and may be related to CD4+ T cell depletion.

**Table 1:** *Ex vivo* pro- and anti-apoptotic molecule expression in HIV-1-infected patient T cells.

Apoptotic factor	Healthy controls		HIV-1-infected patients	
Factor	CD4+ T cells	CD8+ T cells	CD4+ T cells	CD8+ T cells
Bcl-2	1301±62∧	1424±208	1204±56	**953±140** **
Bcl-xL	398±14	464±54	**581±77** *	591±77
c-FLIP	554±101	522±79	528±102	545±129
FADD	915±69	783±64	972±83	785±69
Bid	111±28	115±28	120±16	114±13
Bim	199±24	218±25	**417±64** ***	379±61
Bax	48±13	55±11	**110±16** **	**113±17** *
Bak	96±23	113±24	**171±27** *	**185±18** *

∧ ΔMFI ± standard error.

* P<0.05,

** P<0.02,

*** P<0.005 compared to healthy controls.

**Figure 3:**
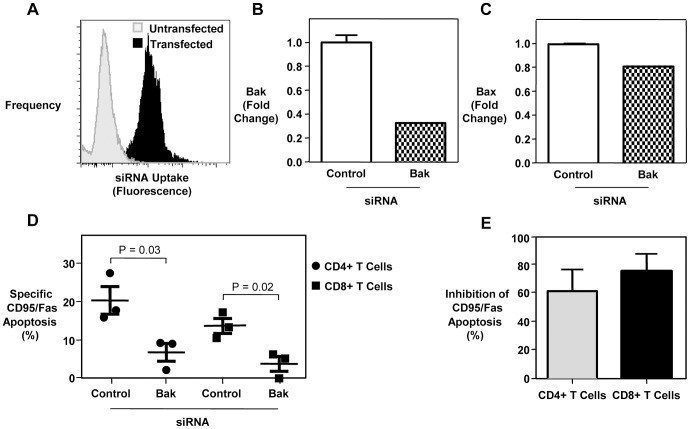
Bak silencing markedly reduces the sensitivity of T cells in chronic HIV-1 infection to CD95/Fas-induced apoptosis. (A) Efficiency of siRNA uptake in T cells transfected with a pool of siRNAs and siGlo fluorescent oligonucleotides (to monitor transfection) compared to an electroporated negative control (no RNA or siGlo). Silencing of (B) Bak and (C) Bax (off-target control) by siRNA sequences targeting Bak in primary PBMC from a representative HIV+ patient measured by quantitative PCR. Expression (mean, SEM) normalized to 18S rRNA is presented as fold change relative to non-targeting control siRNA. (D) CD95/Fas-specific apoptosis of CD4+ T cells and CD8+ T cells in chronic HIV-1 infection following transfection with a pool of siRNAs directed against Bak, relative to a pool of non-targeting siRNAs (negative control). (E) Percent inhibition of CD95/Fas-specific cell death in T cells from chronically HIV-1-infected individuals following transfection with a Bak siRNA pool. Results were obtained from 3 independent experiments with 3 different HIV-1-infected patients.

In order to determine whether *ex vivo* expression of Bak is predicative of cell death, we correlated Bak expression and T cell apoptosis sensitivity. We found a significant positive correlation between Bak expression and CD95/Fas-induced apoptosis of CD4+ T cells and CD8+ T cells, but not with spontaneous apoptosis or AICD (Figure 2C). To confirm the involvement of Bak in CD95/Fas-induced death of T cells, we performed Bak knockdown using siRNA (Figure 3A–B). Targeting of Bak with siRNA resulted in a marked reduction of Bak expression (Figure 3B), with minimal off-target effects (Figure 3C). The introduction of siRNA directed against Bak into T cells from chronically HIV-1-infected patients caused a marked reduction in apoptosis induced by CD95/Fas cross-linking, compared to a control siRNA pool (Figure 3D–E). Therefore, reducing Bak expression drastically decreases the susceptibility of T cells in chronic HIV-1 infection to CD95/Fas-mediated death. These findings demonstrate that Bak is increased in T cells during chronic HIV-1 infection, correlates with CD4+ T cell depletion and is directly involved in CD95/Fas apoptosis.

HIV-1 disease progression is characterized by well-defined changes in the composition of circulating lymphocyte subpopulations, including the gradual loss of naïve and memory T cells [33]. We therefore wanted to determine whether the level of Fas apoptosis susceptibility varies between T cell differentiation states. Indeed, Fas apoptosis sensitivity was higher in CD45RA^−^CD62L^−^ effector memory populations and in terminally differentiated CD45RA^+^CD62L^−^ effector memory T cells (Figure S2A). In contrast, CD45RA^+^CD62L^+^ naïve T cells and CD45RA-CD62L+ central memory populations were resistant to apoptosis (Figure S2A). In line with these data, Bak levels were significantly elevated in effector memory subpopulations, as compared to naïve T cells (Figure S2B). Increased levels of Bak were also observed in central memory T cells, relative to naïve T cells (Figure S2B). However, increased Bak in central memory T cells may be counterbalanced by elevated Bcl-xL expression in this specific subset, thus potentially accounting for the relative resistance of these cells to apoptosis (Figure S2C). Therefore, CD95/Fas-mediated death and Bak expression in CD4+ T cells and CD8+ T cells is associated with the differentiation stage of these cells.

We next examined whether the magnitude of plasma viremia in HIV-1-infected subjects affects the expression levels of Bak in peripheral CD4+ T cells. Total *ex vivo* levels of Bak were significantly increased in CD4+ T cells from ART-treated patients with high-level viremia (≥1000 HIV-1 RNA copies/ml of plasma; Bak MFI = 375±44), as compared to patients with low-level viremia (<1000 HIV-1 RNA copies/ml of plasma; Bak MFI = 253±30) (Figure S3A). In contrast, CD4+ T cells from HIV-1-infected individuals with high viral loads did not exhibit elevated levels of Bax (MFI = 271±56 vs. 296±52) or Bim (MFI = 767±125 vs. 673±29) (Figure S3B–C). Therefore, high levels of HIV-1 in the peripheral blood plasma may result in increased pro-apoptotic Bak expression and this could influence the sensitivity of CD4+ T cells to CD95/Fas-induced death.

### Type I IFN increases Bak expression and CD95/Fas-mediated apoptosis sensitivity in resting and activated T cells

**Figure 4:**
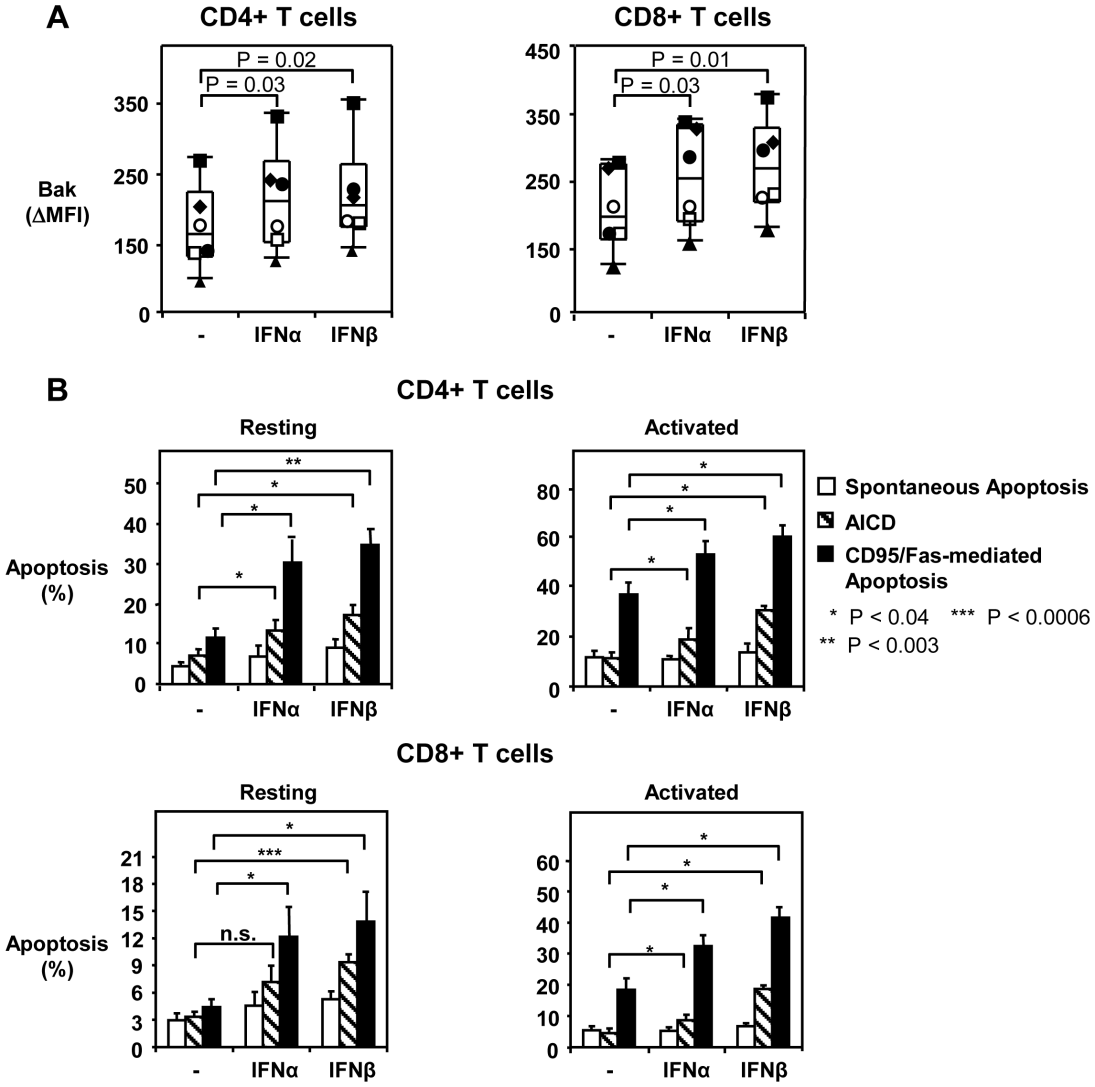
Type I IFN increases Bak expression in T cells from healthy donors and sensitizes them to CD95/Fas-mediated apoptosis. (A) Bak expression shown in CD4+ T cells and CD8+ T cells from healthy individuals after PBMC were untreated or treated with Type I IFN (1000 U/ml) for 72 hours. Each symbol depicts one donor (n = 6). Lines indicate 10% and 90% and the box depicts 25%, median and 75% quantiles. (B) Apoptosis sensitivity of T cells shown following a 72 hour Type I IFN treatment. Cells were stimulated with media alone (spontaneous apoptosis), plate-bound anti-CD3 antibody (AICD) or plate bound anti-CD95 antibody for 14 hours after pre-stimulation with Type I IFN as described in A and apoptosis was measured (n = 5). Bars depict mean ± standard error. P values shown in Panel A and B graphs were calculated by using the Student's t-test for paired samples.

**Figure 5:**
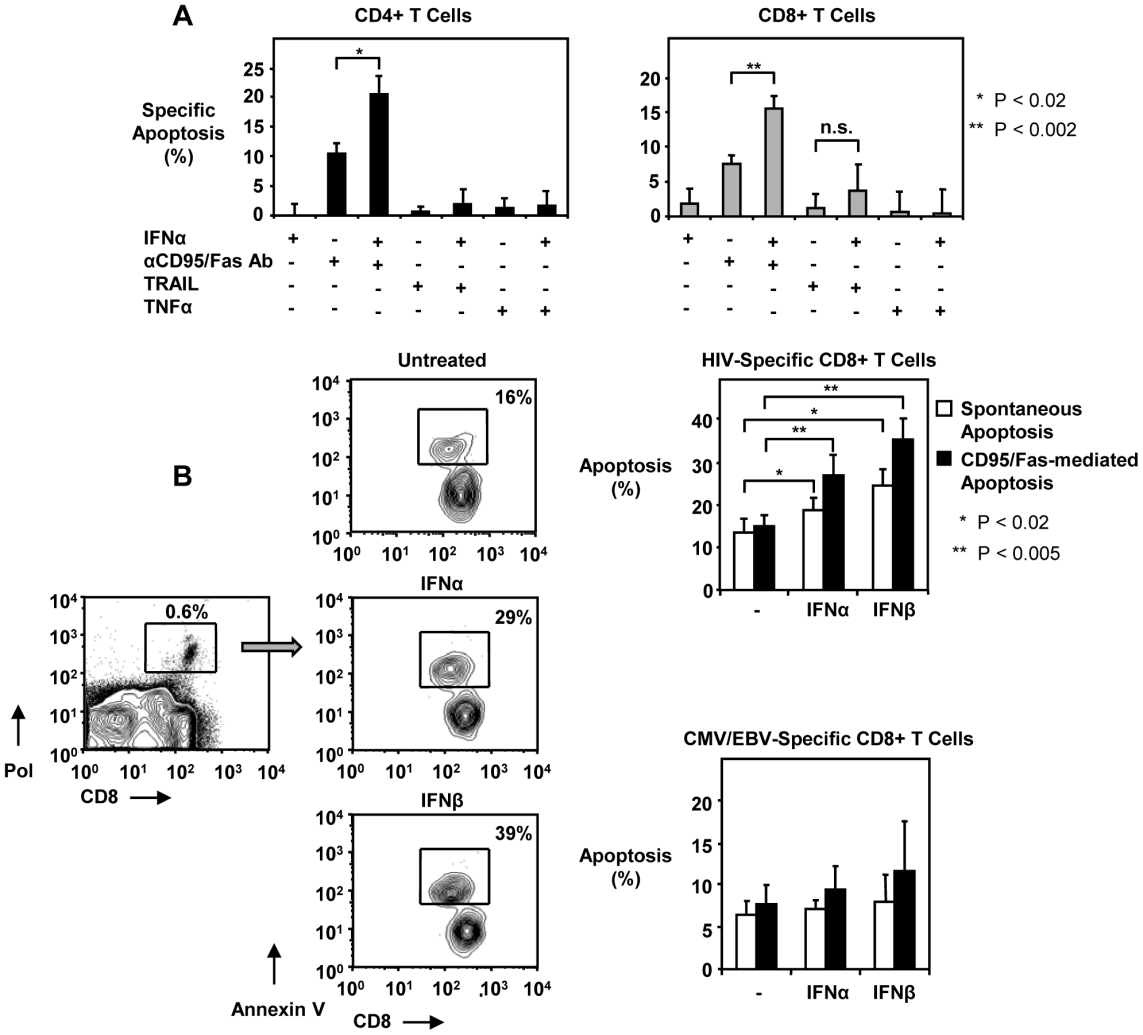
Type I IFN increases the sensitivity of T cells from HIV-1-infected individuals to CD95/Fas-induced death, but not TRAIL- and TNFα-mediated apoptosis. (A) Treatment-specific apoptosis shown for CD4+ T cells (n = 10) and CD8+ (n = 10) T cells pre-treated with IFNα (1000 U/ml) for 72 hours prior to a 14 hour stimulation with plate-bound anti-CD95/Fas antibody, TRAIL (10 ng/ml) or TNFα (10 ng/ml). n.s. = not significant. (B) Representative flow cytometry (left panel) and pooled data (right panel) showing apoptosis sensitivity of HIV-specific CD8+ T cells (n = 7) and CMV/EBV-specific CD8+ T cells (n = 5) after pre-incubation of PBMC with IFNα or IFNβ (1000 U/ml) for 72 hours and subsequent stimulation with plate-bound anti-CD95/Fas antibody for 14 hours. Bars in graphs indicate mean ± standard error. P values shown in graphs were calculated by using the parametric Student's t-test or nonparametric Wilcoxon signed rank test for paired samples.

In order to address the question of what drives Bak overexpression in HIV-1 infection, we examined whether IFNα/β alters Bak levels and increases T cell apoptosis. PBMC from healthy individuals were treated with Type I IFN for 3 days in the absence or presence of CD3-induced activation. Type I IFN significantly increased the expression of Bak in both resting and activated CD4+ T cells and CD8+ T cells (Figure 4A; Figure S4). In addition, both CD4+ T cells and CD8+ T cells cultured in the presence of IFNα exhibited significant upregulation of CD95 expression (Figure S5A–B). Type I IFN did not affect the expression levels of Bcl-2, Bcl-xL, FADD, Bid, Bim or Bax in CD4+ T and CD8+ T cells (Table 2). When we tested T cell apoptosis sensitivity after 3 days of pre-incubation with Type I IFN, we found a 3-fold increase in CD95/Fas-induced apoptosis of resting and activated CD4+ T cells treated with IFNα/β compared to untreated cells (Figure 4B). A similar effect was seen in CD8+ T cells (Figure 4B). While Type I IFN induced slight increases in AICD, it did not enhance the spontaneous death rate of T cells (Figure 4B). As anticipated, a direct correlation was observed between Type I IFN-mediated Bak elevation in CD4+ T cells and increased sensitivity to CD95/Fas-induced death (Figure S5C), but not spontaneous apoptosis or AICD (data not shown). IFN treatment also increased CD4+ T cell and CD8+ T cell apoptosis in HIV-1-infected patients (Figure 5A). In contrast, neither TRAIL- nor TNFα-mediated apoptosis were increased by IFNα treatment (Figure 5A). Additionally, Type I IFN increased the susceptibility of HIV-specific CD8+ T cells, but not CMV/EBV-specific from HIV-1-infected individuals to CD95/Fas-induced death (Figure 5B). However, Type I IFN did not sensitize HIV-specific CD8+ T cells to TRAIL or TNFα-induced apoptosis (Figure S6). Taken together, the above findings indicate that IFNα/β enhances the sensitivity of T cells in HIV-1 infection to CD95/Fas-induced apoptosis, possibly through upregulation of Bak. Type I IFN may also be involved in the survival defect of HIV-specific CD8+ T cells.

**Table 2:** Pro- and anti-apoptotic protein expression in health donor T cells untreated or treated with Type I IFN.

Apoptotic factor	Untreated control		IFNα-treated (72 Hours)	
Factor	CD4+ T cells	CD8+ T cells	CD4+ T cells	CD8+ T cells
Bcl-2	585±21∧	620±40	562±54	585±65
Bcl-xL	290±140	309±129	240±124	242±128
c-FLIP	181±54	187±58	225±80	226±82
FADD	555±179	451±157	502±147	395±117
Bid	351±57	348±55	412±42	396±34
Bim	177±53	206±67	189±37	206±46
Bax	247±34	239±33	281±51	272±53
Bak	178±24	210±25	**217±30** *	**259±30** *

∧ ΔMFI ± standard error.

* P = 0.03 compared to untreated control.

### HIV-1-exposure increases T cell CD95/Fas-mediated apoptosis by inducing Type I IFN

**Figure 6:**
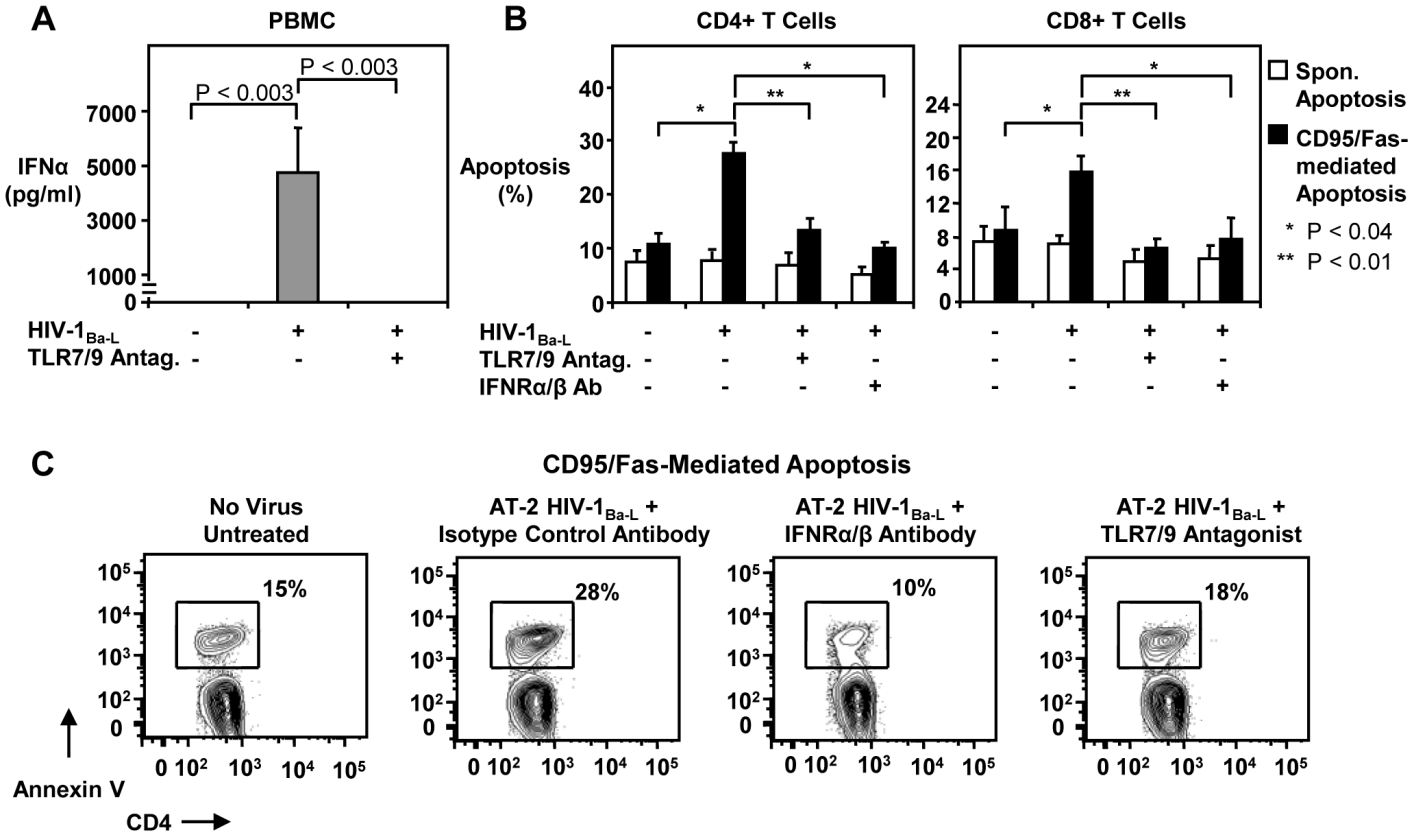
CD95/Fas-induced apoptosis of T cells exposed to HIV-1 is Type I IFN-dependent. (A) TLR7/9 inhibitors inhibit HIV-1_Ba-L_-induced production of IFNα in infected PBMC from healthy donors. PBMC were exposed to 10^5^ TCID_50_/ml HIV-1_Ba-L_ in the presence or absence of a TLR7/9 antagonist for 24 hours (n = 13) and IFNα in supernatants was measured by ELISA. Bars indicate mean ± standard error. P values were calculated by using the Wilcoxon signed rank test for paired samples. (B) HIV-1_Ba-L_ exposure increases CD95/Fas apoptosis of T cells, which is inhibited by a TLR7/9 antagonist and anti-IFNα/β receptor blocking antibodies. Sensitivity to CD95/Fas-induced apoptosis shown for CD4+ T cells and CD8+ T cells from healthy donors following a 72 hour exposure to HIV-1_Ba-L_ in the presence or absence of anti-IFNα/β receptor blocking antibodies or a TLR7/9 antagonist (n = 5). Bars depict mean ± standard error. P values were calculated by using the Student's t-test for paired samples. (C) Representative flow cytometry plots from one out of three healthy donors depicting the sensitivity of CD4+ T cells to CD95/Fas-mediated apoptosis following exposure to non-infectious (AT-2 treated) HIV-1_Ba-L_ for 72 hours in the presence or absence of an isotype control antibody, anti-IFNα/β receptor blocking antibody, and a TLR7/9-specific antagonist. Numbers indicate the percentage of apoptosis.

Because TLR7 and TLR9 recognition of HIV-1 and SIV in pDC triggers Type I IFN production [9], [34], [35], we next evaluated whether HIV-1 exposure of PBMC from healthy individuals would lead to Type I IFN production and a subsequent increase in T cell apoptosis sensitivity. Although pDC are present at very low frequency in the blood, they produce 100 times more Type I IFN per cell than monocytes [36]. We exposed healthy PBMC to virus in the presence or absence of a TLR7/9-specific antagonist and IFNα/β receptor blocking antibodies and analyzed CD95/Fas-mediated apoptosis. PBMC exposed to 10^5^ TCID_50_/ml of HIV-1_Ba-L_ for 24 hours secreted IFNα, and this production was significantly reduced in the presence of a phosphorthioate-based TLR7/9 antagonist (Figure 6A). The TLR antagonistic properties of the phosphorothioate deoxyribose compound that was used does not depend on a specific immunoregulatory DNA sequence [37] and can selectively suppress TLR7/9 activation, with no apparent cross-reactivity involving other TLRs [38]. The mechanism of TLR inhibition may be attributed to the ability of the inhibitor to bind TLR7 and TLR9 specifically [38], or its capacity to physically interact with cognate TLR7/9 ligands [37]. This ultimately prevents receptor triggering, MyD88 activation [39] and downstream signaling.

HIV-1-exposed CD4+ T cells and CD8+ T cells showed significantly increased CD95/Fas-mediated apoptosis compared to unexposed cells (Figure 6B). This death was inhibited by a TLR7/9 antagonist or by blocking the IFNα/β receptor (Figure 6B), suggesting that the enhanced apoptosis sensitivity observed in these cells was Type I IFN-dependent. To exclude the effect of productive viral infection on T cell death in these assays, we used AT-2-inactivated HIV-1. The non-infectious AT-2 HIV-1_Ba-L_ significantly augmented CD95/Fas-induced apoptosis of CD4+ T cells and this effect was abrogated in the presence of either anti-IFNα/β receptor blocking antibodies, or a TLR7/9-specific antagonist (Figure 6C). Thus, triggering of TLR7/9 by HIV-1 elicits the production of Type I IFN which sensitizes T cells to undergo CD95/Fas-mediated apoptosis.

To determine whether HIV-1-mediated induction of CD95/Fas apoptosis in CD4+ T cells is largely attributed to productive infection or bystander effects of the virus, we cultured purified CD4+ T cells and PBMC from the same healthy donors in parallel with HIV-1 for 72 hours, followed by a 14 hour stimulation with immobilized anti-CD95 antibodies. Prior to induction of CD95/Fas-induced apoptosis, Bak levels were quantified and the frequency of CD95-expressing lymphocytes was determined in the above cell populations. Notably, HIV-1 did not directly induce Fas apoptosis sensitivity, Bak upregulation or an increase in the frequency of CD95-expressing CD4+ T cells in purified CD4+ T cell cultures (Figure S7A–C). However, CD95/Fas apoptosis sensitivity, Bak levels and the proportion of CD95-expressing lymphocytes were markedly elevated in the CD4+ T cell population present in HIV-1-exposed PBMC (Figure S7A–C). These data suggest that increased CD95/Fas-mediated death of CD4+ T cells is an indirect consequence of HIV-1 exposure and not a direct viral cytopathic effect.

### Type I IFN-induced gene expression is elevated in HIV-1-infected patients and correlates with T cell apoptosis sensitivity, CD4+ T cell loss and increased plasma viremia

**Figure 7:**
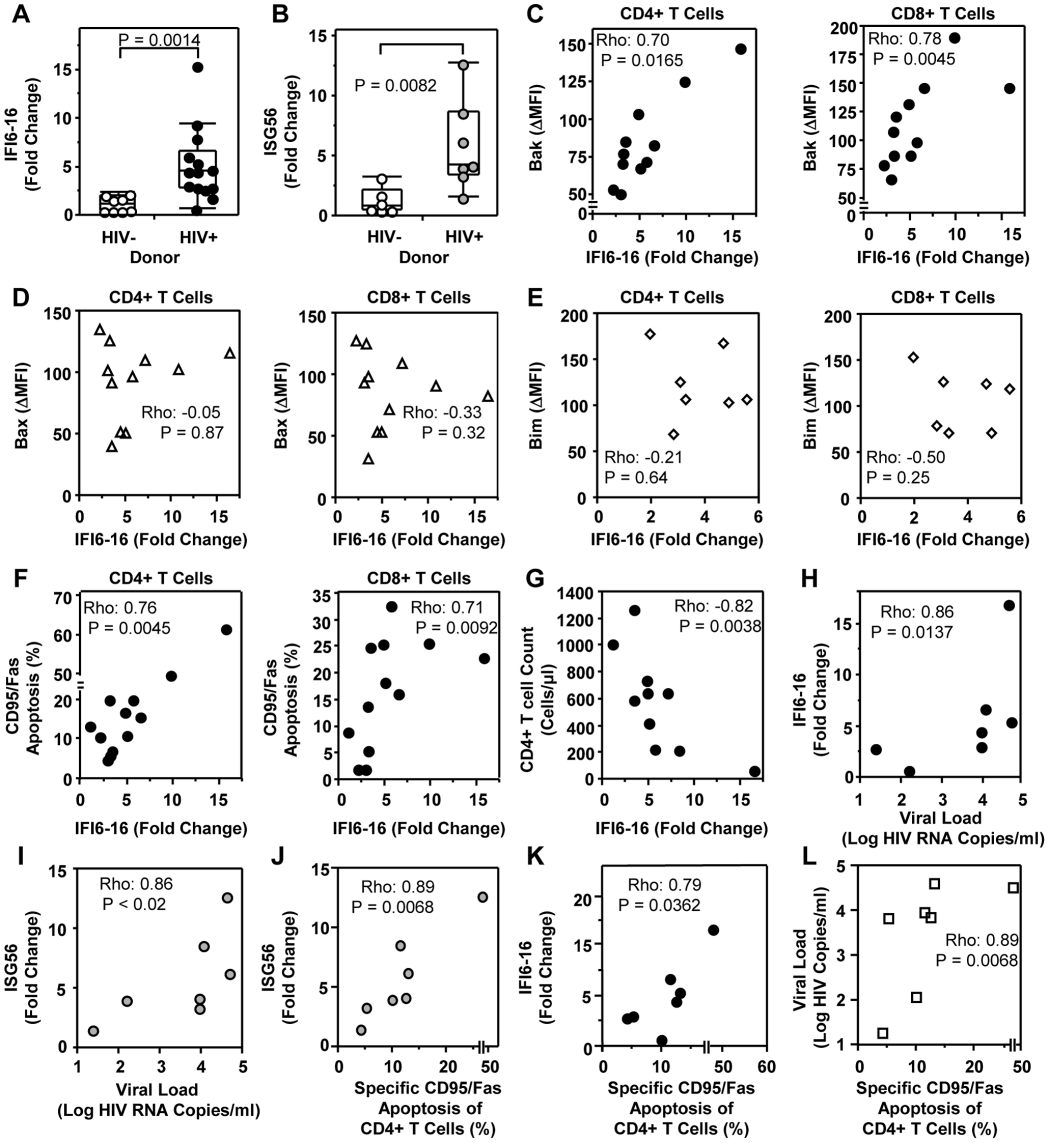
IFNα-induced gene expression is increased in HIV-1 infection and correlates with Bak expression, CD95/Fas-induced T cell apoptosis and CD4+ T cell loss. (A) *Ex vivo IFI6-16* and (B) *ISG56* levels as measured by real-time RT PCR are significantly increased in HIV-1-infected patients (n = 7–14) compared to healthy donors (n = 6–8). P values were calculated by using the Wilcoxon signed rank test for unpaired samples. Spearman's rho correlations shown between *IFI6-16* expression in PBMC and (C) *ex vivo* T cell Bak expression (n = 11), (D) *ex vivo* T cell Bax expression (n = 11) (E) *ex vivo* T cell Bim expression (n = 7) (F) *ex vivo* CD95/Fas-mediated T cell apoptosis sensitivity (n = 12) and (G) CD4+ T cell counts (n = 10) in ART-naive HIV-1-infected individuals. Correlations are shown for ART-naïve HIV-1-infected patients (n = 7) between plasma viral load and (H) IFI6-16 mRNA levels, (I) ISG56 mRNA levels. Spearman's rho correlations depicted between CD95/Fas-specific apoptosis of CD4+ T cells and (J) *ISG56* expression, (K) *IFI6-16* expression and (L) plasma viral load in ART-naïve HIV-1 patients.

To directly examine the relationship between Type I IFN, apoptosis sensitivity and CD4+ T cell counts in HIV-1-infected patients, we analyzed mRNA expression levels of the Type I IFN-induced genes, *IFI6-16* and *ISG56* in ART-naïve chronic HIV-1-infected patients. Both IFI6-16 and ISG56 mRNA levels were increased in PBMC from HIV-1-infected patients relative to healthy donors (Figure 7A–7B). *IFI6-16* levels directly correlated with *ex vivo* Bak expression in CD4+ T cells and CD8+ T cells (Figure 7C) and with CD95/Fas-mediated apoptosis sensitivity (Figure 7F; Figure 7K). Similar correlations were found with *ISG56* (Figure 7J and data not shown). *IFI6-16* levels did not correlate with Bax (Figure 7D) or Bim (Figure 7E) expression in *ex vivo* CD4+ T cells and CD8+ T cells from ART-naïve HIV-1-infected patients. Furthermore, *IFI6-16* expression was negatively correlated with CD4+ T cell counts in HIV-1-infected patients with detectable viremia (Figure 7G). Both *IFI6-16* and *ISG56* expression directly correlated with plasma viral load in HIV-1-infected ART-naïve patients (Figure 7H–7I). As expected, plasma HIV-1 viremia strongly correlated with the specific death of CD4+ T cells triggered by CD95/Fas cross-linking (Figure 7L). These results suggest that viremia drives Type I IFN production in chronic HIV-1 disease, which in turn increases Bak expression and apoptosis sensitivity, leading to accelerated rates of T cell depletion.

### The association between plasma Type I IFN and viremia differs in acute and chronic SIV infection

**Figure 8:**
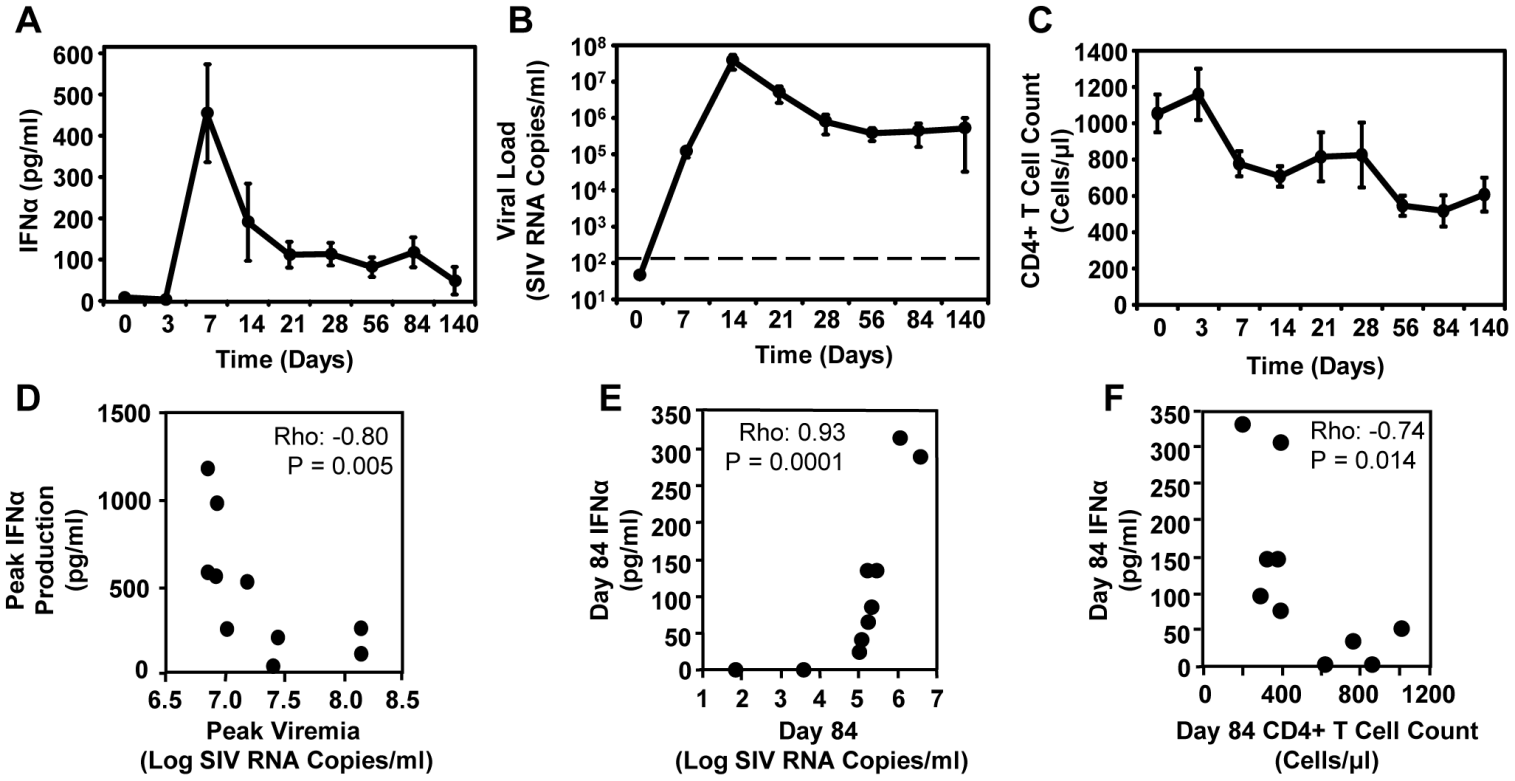
Contrasting relationships between viral loads and Type I IFN levels during acute and chronic SIV infection. (A) Plasma IFNα during pathogenic SIV infection as measured by a modified Luminex assay. (B) Plasma SIV viral load was determined by PCR (dashed line indicates assay detection limit) (C) CD4+ T cell counts during the acute and chronic stages of SIV infection. Spearman's rho correlations shown between plasma IFNα levels and viral loads during (D) acute and (E) chronic SIV infection of rhesus macaques. (F) Spearman's rho correlations shown between IFNα expression and CD4+ T cell counts in chronic SIV infection. Measurements are shown for SIV-infected rhesus macaques (n = 10).

In order to investigate the protective or pathogenic roles of Type I IFN in an animal model of HIV-1 infection, we examined the relationship between plasma levels of IFNα (Figure 8A), viral loads (Figure 8B) and peripheral CD4+ T cell counts (Figure 8C) during acute and chronic SIV infection in rhesus macaques. We found that IFNα peaks early (day 7) during acute infection (Figure 8A) and precedes the peak plasma viral load (day 14; Figure 8B). These SIV-infected animals exhibited a typical viral load profile (Figure 8B) and CD4+ T cell depletion kinetics (Figure 8C). Importantly, we found that the association of IFNα production with viremia was different during acute and chronic infection. Whereas peak IFNα levels were negatively associated with peak viremia during acute SIV infection (Figure 8D), IFNα levels and plasma viral loads were positively correlated during chronic infection in the same animals (Figure 8E). Similar to HIV-1-infected patients, we found that plasma IFNα levels were inversely correlated with CD4+ T cell counts during chronic SIV infection (Figure 8F). These data indicate that although elevated IFNα secretion during acute infection may suppress peak viral replication, during chronic SIV infection, viremia-induced IFNα at low levels drives CD4+ T cell decline and the possible loss of viral control.

## Discussion

A hallmark of HIV-1 infection is the gradual loss of both CD4+ T cells and CD8+ T cells that has been associated with disease progression [40]. Although multiple factors may contribute to T cell depletion, exaggerated apoptosis of T cells is most closely related to CD4+ T cell loss [41]. Despite the large body of literature that has emerged concerning the death pathways responsible for T cell destruction during HIV-1 infection, the relative contributions of TNF receptor family members are a matter of debate. The increased susceptibility of CD4+ T cells from HIV-1-infected patients to CD95/Fas-mediated apoptosis has been previously established [5], [6], [7], [27], [28], [29], [42]. The importance of the CD95/Fas pathway is further supported by blocking FasL in SIV-infected macaques, which inhibits CD4+ T cell apoptosis and preserves CD4+ T cell counts following acute SIV infection [43]. Some studies have suggested a role for TRAIL [44], [45], [46], [47] and TNF [48], [49], whereas other reports indicate that direct engagement of their receptors has a much weaker effect or does not increase T cell apoptosis [48], [50], [51].

Apoptosis may also impair anti-HIV immunity. HIV-specific CD8+ T cells are susceptible to CD95/Fas-induced apoptosis [7], but their sensitivity to TRAIL and TNFα is currently unknown. We find that *ex vivo* T cells from HIV-1-infected patients are very sensitive to CD95/Fas-mediated apoptosis, but not to TNFα or TRAIL-induced death. Furthermore, consistent with our previous findings [5], [7], CD95/Fas stimulation markedly enhanced the susceptibility of HIV-specific CD8+ T cells to undergo apoptosis. In contrast, HIV-specific CD8+ T cells were not primed for apoptosis in response to stimulation with TRAIL or TNFα. These findings further suggest that CD95/Fas is the TNF receptor family member that is the critical mediator of CD4+ T cell and HIV-specific CD8+ T cell depletion during HIV-1 infection. The susceptibility of T cells in HIV-1 disease to CD95/Fas-induced death and relative resistance to TRAIL and TNFα-mediated killing may be attributed to differences in signaling induction of apoptosis between these pathways, including death receptor and ligand expression or the extent of cross-linking, the presence of death domains [52], as well as regulation of their adapter molecules [53], and preferential usage of apoptotic mitochondrial mediators such as Bak and Bax [54]. Elucidation of these exact differences is the subject of ongoing studies.

Because direct viral cytopathic effects alone cannot account for the increased death of peripheral T cells in HIV-1 infection, it is important to determine what factors control bystander CD95/Fas T cell apoptosis sensitivity. We and others have previously shown that the level of CD95 expression on primary T cells from HIV-1-infected patients is not predictive of apoptosis sensitivity [7], [55]. This suggests that intracellular signaling pathways are altered in these cells. In this study, we found that other major signaling molecules that associate with the CD95/Fas pathway [56], including c-FLIP and FADD were not increased in T cells from HIV-1-infected patients. Therefore, signaling proximal to CD95 alone is not likely to account for this enhanced T cell apoptosis sensitivity.

Mitochondria act as an amplification loop for CD95/Fas-mediated apoptotic signaling and triggering of CD95 leads to activation of Bcl-2 homology 3 (BH_3_)-only proteins such as Bid and Bim, which act to compromise mitochondrial integrity either directly by activating Bak/Bax or indirectly by neutralizing Bcl-2 and Bcl-xL [57], [58]. We have previously demonstrated that co-localization between CD95/Fas and mitochondria occurs early in HIV-specific CD8+ T cells upon death-receptor triggering [59]. A role for mitochondria in this apoptotic process is further supported by the reduced expression of anti-apoptotic Bcl-2 family members in T cells during HIV-1 infection [28], [30] and our current study confirmed this. However, little is known about the pro-apoptotic proteins of the Bcl-2 family. Our data indicated that *ex vivo* Bak, Bax and Bim were significantly increased in T cells from HIV-1 infected individuals. Interestingly, only Bak was found to be inversely correlated with the loss of CD4+ T cells *in vivo*. Bak levels were also directly correlated with CD95/Fas apoptosis of T cells in HIV-1-infected patients and inhibiting Bak abrogated this cell death. High levels of Bak expression and elevated CD95/Fas-induced apoptosis of T cells were predictive of CD4+ T cell decline *in vivo* suggesting a pathogenic role for this death pathway. We also observed that effector memory T cells expressed abnormally high levels of Bak and were highly sensitive to Fas apoptosis, as compared to naïve cells. The relative resistance of central memory cells to apoptosis, despite elevated Bak expression may be attributed to significantly increased levels of Bcl-xL expressed in this specific subset. Increased apoptosis has been proposed as a major defect that affects both the differentiation and effector function of T cells during chronic HIV-1 infection [60]. Therefore, understanding how the critical balance of specific pro- and anti-apoptotic factors changes as a function of differentiation status may potentially permit us to elucidate the molecular pathways that control the depletion of particular subpopulations in HIV-1 disease. Interestingly, in chronic SIV infection, higher Bak expression has been demonstrated in CD4+ T cells from the lymph nodes of moderate progressors as compared to slow progressors [61]. Our studies have thus revealed a novel role for Bak upregulation in CD95/Fas T cell apoptosis and peripheral T cell depletion during HIV-1 infection.

The mechanisms underlying the upregulation of Bak in T cells and the consequent increase in the death rate of T cells during HIV-1 and SIV infection are currently unknown. We find that Type I IFN induced Bak overexpression in T cells derived from healthy individuals and this was accompanied by increased sensitivity to CD95/Fas-mediated apoptosis. This suggested that in HIV-1 seropositive patients, Type I IFN may be responsible for accelerated T cell apoptosis and depletion. To test this hypothesis, we used IFN-stimulated gene (ISG) expression in PBMC from patients as a marker of *in vivo* IFNα exposure. ISGs are excellent surrogate indicators of IFN activity because they can be induced by very low levels of IFNα [23]. In HIV-1-infected patient PBMC, IFNα-stimulated gene expression was elevated and positively correlated with high Bak levels in T cells. Furthermore, IFNα-induced gene expression correlated directly with the sensitivity of T cells to CD95/Fas-induced death and inversely with absolute CD4+ T cell counts. ISG expression directly correlated with viremia, suggesting that viral loads in HIV-1-infected individuals drive Type I IFN production and this leads to accelerated T cell apoptosis and loss. This is further supported by the finding that patients with higher viral loads had higher Bak expression in CD4+ T cells. Accordingly, we also show that HIV-1 can induce CD95/Fas-induced T cell apoptosis in a TLR7/9- and Type I IFN-dependent manner and this effect does not require virus replication.

Although CD95 expression is not a predictive correlate of apoptosis sensitivity, it is a necessary component involved in the delivery of the Fas apoptosis signal. We found that Type I IFN upregulated the expression levels of CD95 on healthy donor T cells. These findings, coupled with our observations that HIV-1 exposure markedly increased the frequency of CD95-expressing CD4+ T cells in PBMC, but not purified CD4+ T cells further suggests that Fas apoptosis sensitivity of T cells in HIV-1 disease is a bystander effect of the virus which is likely mediated by IFN production by a cell population other than the T lymphocytes, most likely pDCs [9], [34], [35], [36]. IFNα may have additional deleterious effects by inducing inhibitory receptors such as programmed cell death-1 (PD-1) on T cells [62]. Subsequent studies are required to elucidate the direct or indirect role of IFNα-mediated PD-1 expression on the decline of T cells during chronic viral infections. Our results indicate that during chronic HIV-1 infection, the virus elicits Type I IFN production which upregulates CD95 levels and elevates Bak expression, thereby increasing the susceptibility of T cells to CD95/Fas apoptosis. This aberrant apoptosis induction ultimately results in CD4+ T cell loss and impairment of HIV-specific CD8+ T cells.

The potential deleterious effect of Type I IFN is further supported by SIV studies which show that non-pathogenic SIV-infections exhibit reduced IFNα compared to pathogenic SIV-infections [9], [23], [63], and this may be associated with reduced T cell apoptosis [64], [65]. Previous studies of pathogenic SIV infection have shown that plasma IFNα peaks and returns to undetectable levels early during acute infection [23], [66]. We show here that SIV disease is associated with persistent systemic IFNα generation that can be detected during the acute and chronic stages of infection. Our results demonstrate that SIV-infected rhesus macaques with higher levels of peak IFNα exhibited a significantly lower peak viral burden during acute infection, suggesting a potential beneficial anti-viral effect of the Type I IFN response. However, at the chronic stage of SIV infection in these same animals, higher viremia resulted in elevated plasma IFNα, suggesting that the virus drives IFNα production and the beneficial effect of IFN is largely lost. Our interpretations of these correlations are strongly supported by two very recent studies showing that persistent Type I IFN production in chronic (Clone 13) lymphocytic choriomeningitis virus (LCMV) infection is causatively associated with the loss of viral control and this effect is CD4+ T cell-dependent [67], [68]. In contrast, Type I IFN signaling blockade during acute (Armstrong) LCMV infection results in severely impaired viral clearance [67]. Although these reports suggest that Type I IFN may have stage-specific opposing effects during viral infections, the impact of the immunoprotective and immunopathological effects of these cytokines during the acute and chronic stages of single viral infection over time is currently unknown [69]. Our investigation addresses this question in part for pathogenic SIV infection through examination of the longitudinal relationships between Type I IFN production, CD4+ T cell decline and viral load.

The finding that IFNα in chronic SIV infection is detrimental to the host was suggested by the observation that higher plasma IFNα was associated with loss of CD4+ T cells in these monkeys. One explanation for the contrasting effect of IFNα during acute and chronic SIV infection may be the different levels of IFN that are produced. Thus, the beneficial versus the harmful effect of IFNα may be determined by cytokine concentration, with high concentrations suppressing virus replication and persistent low levels leading to T cell loss. This is supported by studies which demonstrate that administration of high dose IFNα or pharmacologic induction of a robust Type I IFN response enhances antiviral immunity [70], [71], while continuous low-dose IFNα treatment drives strong lymphopenia in chronically SIV-infected monkeys [70]. HIV-1 can clearly induce Type I IFN and this could explain the positive correlation between IFNα and viral load during chronic infection. An alternative explanation, in light of the observation that Type I IFN primes HIV-specific CD8+ T cells for apoptosis, is that increased IFN impairs both the function and survival of these cells, contributing to the loss of viral control.

In conclusion, our data support a scenario in which the induction of IFNα/β during chronic HIV-1 infection exerts pathogenic instead of protective effects on host immunity by upregulating CD95 and Bak expression and sensitizing CD4+ T cells and CD8+ T cells to Fas-mediated apoptosis, but not to TRAIL or TNFα-induced death. Our SIV studies suggest that although during acute SIV infection, elevated Type I IFN levels may contribute to lower peak viremia, this is not the case during chronic infection where viral loads seem to drive elevated IFNα levels. Continued production of Type I IFN during chronic HIV-1 disease may compromise CD4+ T cell survival, in addition to impairing HIV-specific CD8+ T cells. Therefore, blocking the activity of Type I IFN or its production with TLR antagonists may be a useful strategy to inhibit CD4+ T cell loss and enhance HIV-specific immunity.

## Materials and Methods

### Ethics statement

Peripheral blood was collected from individuals following Drexel University College of Medicine Institutional Review Board (IRB) approval and obtaining written informed consent. Rhesus macaques (Macaca mulatta) were housed at BIOQUAL, Inc. (Rockville, MD), in accordance with the standards of the American Association for Accreditation of Laboratory Animal Care. The protocol was approved by the BIOQUAL's Institutional Animal care and Use Committee under OLAW Assurance Number A-3086-01. Bioqual is IAAALAC accredited and procedures were carried out in accordance with the recommendations of the Weatherall report.

### Human subjects

All of the patients were HIV-1 positive for at least 1 year (range 1–30 years); median CD4 count was 432 cells/µl (range 10–1,551 cells/µl); median CD8 count was 980 cells/µl (range 224–2,565 cells/µl); median viral load was 325 RNA copies/ml blood (range <20–978,190 copies/ml blood); 66 patients were on antiretroviral therapy (ART). HIV-1 viral loads (HIV RNA copies/ml plasma) were determined using COBAS AmpliPrep/COBAS TaqMan HIV-1 Test, v2.0 (Roche Diagnostics, Indianapolis, IN) with an assay range from 20–10,000,000 HIV RNA copies/ml. IFNα-induced gene expression studies were conducted with ART-untreated HIV-1-infected patients (ART naïve). Control samples were obtained from HIV-1 seronegative age-matched healthy individuals. All assays were performed on freshly isolated PBMC from HIV-1-infected and healthy individuals.

### Flow cytometry

PBMC were freshly isolated from heparinized venous blood by centrifugation over a Ficoll-Hypaque gradient (Amersham Pharmacia Biotech, Uppsala, Sweden). HIV-specific, CMV-specific and EBV-specific CD8+ T cells were detected using tetramers of HLA class I A*0201 loaded with either HIV-Gag p17 77–85 (SLYNTVATL), HIV-Pol 476–484 (ILKEPVHGV), CMV p65 495–503 (NLVPMVATV) or EBV 280–288 (GLCTLVAML) peptide. To analyze the apoptosis sensitivity of PBMC, cells were stained with Annexin V Cy5.5/anti-CD8 PE-Texas Red (Caltag, Burlingame, CA)/anti-CD4 FITC/anti-CD3 Pacific Blue/HIV- or CMV-specific tetramer APC. Annexin V and all other antibodies were purchased from BD Biosciences (San Diego, CA). Briefly, 10^6^ cells were stained with tetramers and antibodies in FACS wash (HBSS (Cellgro, Herndon, VA), 3% horse serum (Life Technologies, Carlsbad, CA), 0.02% NaN_3_) for 30 minutes on ice; washed with FACS wash and fixed with 1% paraformaldehyde. When Annexin V staining was performed, 2.5 mM CaCl_2_ was included in all steps. Intracellular levels of pro- and anti-apoptotic proteins were measured directly *ex vivo* in PBMC or following a 3 day incubation in the presence or absence of 1000 U/ml IFNα/β ± anti-CD3 antibody (plates coated with 0.1 µg/ml). Following Live/Dead (Invitrogen) and surface staining with anti-CD8 PE-Texas Red/anti-CD4 FITC/anti-CD3 Pacific Blue, cells were permeabilized with cytotofix/cytoperm buffer (BD Biosciences) and intracellular staining was performed for 1 hour with fluorochrome conjugated antibodies (anti-Bcl-2 and anti-Bcl-xL antibodies, BD Biosciences), or specific primary antibodies (anti-FADD, Biovision, Mountain View, CA; anti-Bid, Epitomics, Burlingame, CA; anti-Bim, Millipore, Billerica, MA; anti-Bax, Abgent, San Diego, CA; anti-Bak, Epitomics) and appropriate isotype controls, followed by a 1 hour incubation with a secondary antibody (PE-conjugated anti-rat IgG or anti-rabbit IgG, Southern Biotechnologies). Samples were collected on a FACSAria (BD Biosciences) and analyzed using FlowJo software (Treestar, San Carlos, CA). Mean fluorescence intensity (MFI) of intracellular protein expression as expressed as delta (Δ) MFI indicates: MFI of specific antibody – MFI of isotype control.

### Apoptosis assays

To determine the sensitivity of T cells to apoptosis stimuli, PBMC (10^6^ cells/ml) from HIV-1-infected individuals or healthy donors were stimulated in complete RPMI (RPMI 1640/10% heat-inactivated fetal bovine serum/2 mM L-glutamine/100 U/ml penicillin/100 µg/ml streptomycin sulfate, Cellgro, Manassas, VA) at 37°C in 5% CO_2_, in the presence or absence of plate-bound anti-CD95 monoclonal antibody (plates coated with 5 µg/ml, CH11, Millipore), soluble SuperKiller (cross-linked) TRAIL (10 and 100 ng/ml, Alexis Biochemicals, Lausen, Switzerland) or TNFα (10 and 100 ng/ml, R & D Systems, Inc., Minneapolis, MN) for 14 hours, harvested and stained. For IFNα/β apoptosis sensitization studies, PBMC from healthy and HIV-1-infected individuals were first stimulated with human IFNα or IFNβ (1000 U/ml; PBL, Piscataway, NJ) for 3 days in the presence or absence of plate-bound anti-CD3 antibody (plates coated with 0.1 µg/ml OKT3) at 37°C in 5% CO_2_, before apoptosis sensitivity was evaluated by re-stimulating the cells with anti-CD95 monoclonal antibody (plates coated with 5 µg/ml), anti-CD3 monoclonal antibody (plates coated with 5 µg/ml), TRAIL (10 ng/ml) or TNFα (10 ng/ml). Plates were coated with antibodies as previously described [5]. Cells were then stained and fixed for flow cytometry. Specific apoptosis was calculated using the following formula: [(percentage of induced apoptosis - percentage of spontaneous apoptosis)/(100 - percentage of spontaneous apoptosis)]×100.

### Bak small interfering RNA knockdown in HIV-1-infected patient samples

PBMC from chronically HIV-1-infected patients were isolated by Ficoll-Hypaque gradient centrifugation. Knockdown of Bak expression was achieved through siRNA transfection by electroporation using a Gene Pulser XCell (BioRad). PBMC (7.5–8×10^6^ cells) were washed and resuspended in 300 µl of Opti-MEM (Life Technologies) in a 2-mm cuvette and pulsed (square wave, 500 V, 1 ms) with 1 nmol of siRNA (ON-TARGET Non-Targeting pool or Bak ON-TARGETplus SMARTpool, Dharmacon). The siRNA pools consist of a mixture of 4 siRNAs that are pre-designed to reduce off-target effects by up to 90% [72]. Knockdown efficiency in HIV-1 patient PBMC following siRNA transfection was determined by real-time quantitative PCR with Taqman gene expression assays (Applied Biosystems, Foster City, CA) for Bak (assay Hs00832876_g1), Bax (assay Hs00180269_m1) which served as a negative control for knock-down specificity, and 18S rRNA (assay Hs03928985_g1) which served as a loading and normalization control. Transfection efficiency was assessed with siGlo fluorescent oligonucleotides (Dharmacon). Following a 72 hour incubation period, cells were left unstimulated or stimulated with plate-bound anti-CD95 monoclonal antibody for 14 hours and assayed for apoptosis as described above. CD95/Fas-induced specific apoptosis was calculated by subtracting the level of spontaneous cell death and apoptosis caused by electroporation alone.

### PBMC and CD4+ T cell stimulations with infectious and non-infectious HIV-1

A high-titer virus stock was produced following a 3 day cell-associated infection of PM1 cells with HIV-1_Ba-L_. The infectious cell-free virus present in the culture supernatant was treated with 250 µM aldrithiol-2 (AT-2; Sigma Aldrich, St. Louis, MO) for 1 hour at 37°C [73], concentrated and the AT-2 HIV-1 stock was tested for infection. CD4+ T cells were isolated from whole blood by negative selection using the RosetteSep system (STEMCELL Technologies Inc., Vancouver, BC). Purity was >96%, as determined by flow cytometry. Cells were cultured in complete RPMI at a density of 10^6^ cells/ml/well and pre-treated for 1 hour with 5 µM of a TLR7/9-specific antagonist (13mer phosphorothioate deoxyribose inhibitor that we have previously characterized [74]); Invitrogen Corporation, Carlsbad, CA), 10 µg/ml of an anti-IFNα/β receptor blocking antibody (Millipore), or an isotype control (eBioscience); 7×10^4^–10^5^ TCID_50_/ml of infectious HIV-1_Ba-L_ or an equivalent p24 amount of AT-2 HIV-1 was then added for 3 days before the cells were stimulated with anti-CD95/Fas-antibodies for 14 hours. Percentages of apoptotic cells were determined by flow cytometry as described above. IFNα present in supernatants was measured at 24 hours following virus exposure using the Human Interferon Alpha Multi-Subtype ELISA Kit (PBL).

### PCR analysis for Type I IFN-induced gene expression

Total RNA was extracted from the freshly isolated PBMC of HIV-1-infected individuals and healthy donors. The RNA was reverse transcribed into complementary DNA which was then subjected to real-time PCR using gene-specific primers for *IFI6-16* (5′-cctgctgctcttcacttgca-3′ and 5′-ccgacggccatgaaggt-3) and *ISG56* (5′-ctggactggcaatagcaagct-3′ and 5′-gagggtcaatggcgttctga-3′) as described previously [26], [75]. IFNα-stimulated gene expression levels were normalized to β-actin controls and results were calculated for each HIV-1-infected patient as a fold change in gene expression, relative to healthy donors using the delta-delta Ct method of analysis.

### Plasma IFNα measurements in SIV-infected rhesus macaques

Macaques were inoculated intravenously or intrarectally with 100 ID_50_ of SIV_mac251_ and plasma IFNα levels were measured by Luminex as previously described [76] with monoclonals MMHA-11 and MMHA-2 (PBL) used as capture and detector antibodies, respectively. Viral load was determined by TaqMan RNA RT-PCR assay with a sensitivity of 200 SIV RNA copies/ml of blood (Applied Biosystems). Absolute CD4+ T cell numbers were determined by flow cytometry.

### Statistical analysis

Statistical analysis was performed using the Shapiro-Wilk W test for normality, Student's t-test and nonparametric Wilcoxon signed-rank test for paired and unpaired samples. Parametric (Pearson's r) and nonparametric (Spearman's rho) statistics were used for measurements of correlation. Analyses were performed with the JMP program (SAS, Cary, NC). P values<0.05 were considered to be statistically significant.

## Supporting Information

Figure S1 **Total levels of Bak, Bax and Bim are elevated in CD4+ T cells from HIV-1-infected patients, relative to healthy donors.** Representative primary data depicting mean fluorescence intensity (MFI) of total (A) Bak, (B) Bax and (C) Bim from *ex vivo* CD4+ T cells in an HIV-1-infected donor, as compared to healthy subject.
(TIF)

Figure S2 **Apoptosis sensitivity of T cells in HIV-1 disease depends on the cellular differentiation state and the balance of pro- and anti-apoptotic molecules.** (A) Spontaneous and CD95/Fas-mediated apoptosis of memory subpopulations of CD4+ T cells and CD8+ T cells from HIV-1-infected individuals. Levels of total (B) Bak (n = 17) and (C) Bcl-xL (n = 14) in memory subpopulations of CD4+ T cells and CD8+ T cells from HIV-1-infected donors. Each data point represents an individual HIV-1-infected patient. P values were calculated by using the Student's t-test for paired samples.
(TIF)

Figure S3 **Bak levels are increased in HIV-1-infected donors with high viral loads, relative to patients with low viral loads.** Levels of total (A) Bak (low: n = 10; high: n = 7), (B) Bax (low: n = 13; high: n = 11) and (C) Bim (low: n = 12; high: n = 7) in HIV-1-infected individuals with low-level (<1000 HIV RNA copies/ml) and high-level viremia (≥1000 HIV RNA copies/ml). P values for pro-apoptotic molecules were calculated by using the Student's t-test for unpaired samples.
(TIF)

Figure S4 **IFNβ upregulates Bak expression in activated T cells from healthy donors.** Bak expression shown in CD4+ T cells and CD8+ T cells from healthy donors after PBMC were activated with plate-bound anti-CD3 antibody and untreated or treated with IFNβ (1000 U/ml) for 72 hours. Each filled circle represents one healthy donor (n = 8). Lines indicate 10% and 90% and the boxes depict 25%, median and 75% quantiles. P values were calculated by using the Student's t- test for paired samples.
(TIF)

Figure S5 **Type I IFN increases CD95 expression on healthy donor T cells and induces Bak upregulation that is directly correlated with CD95/Fas apoptosis sensitivity.** CD95 expression shown on healthy donor (A) CD4+ T cells and (B) CD8+ T cells after PBMC were untreated or treated with IFNα (1000 U/ml) for 72 hours. Each filled circle represents one donor (n = 6). P values were calculated by using the Student's t-test for paired samples. (C) Pearson's correlation shown between CD4+ T cell Bak expression and CD95/Fas apoptosis sensitivity of activated CD4+ T cells following a 72 hour treatment of healthy donor PBMC with IFNβ (1000 U/ml).
(TIF)

Figure S6 **Type I IFN does not sensitize HIV-specific CD8+ T cells to TRAIL or TNFα-mediated apoptosis.** PBMC were untreated or treated with IFNα (1000 U/ml) for 72 hours. Cells were then unstimulated or cultured with TRAIL (10 ng/ml) or TNFα (10 ng/ml) for 14 hours. Flow cytometric measurements of Annexin V expression were performed on Gag tetramer positive CD8+ T cells. Flow cytometry plots for one representative chronically HIV-1-infected subject are shown.
(TIF)

Figure S7 **HIV-1 exposure differentially affects Fas apoptosis sensitivity, Bak expression and the frequency of CD95+ CD4+ T cells in PBMC versus purified CD4+ T cells.** (A) Representative flow cytometry plots depicting spontaneous death and CD95/Fas-mediated apoptosis of purified CD4+ T cells or CD4+ T cells present in PBMC cultures from the same donor. Cells were exposed to HIV-1_Ba-L_ for 72 hours and were subsequently left unstimulated or stimulated with solid-phase anti-CD95/Fas antibodies for 14 hours (B) Bak expression and (C) frequency of CD95-expressing CD4+ T cells in purified CD4+ T lymphocyte and PBMC cultures from one donor that were unexposed or exposed to 7×10^4^ TCID_50_/ml of HIV-1_Ba-L_ for 72 hours. Results are representative of 2 independent experiments performed with 2 different healthy donors.
(TIF)
