# Supplementary figures and images for: Type I Interferon Upregulates Bak and Contributes to T Cell Loss during Human Immunodeficiency Virus (HIV) Infection
Source: PLoS Pathog. 2013 Oct 10;9(10):e1003658. doi: 10.1371/journal.ppat.1003658 (PMC3795023; doi:10.1371/journal.ppat.1003658)

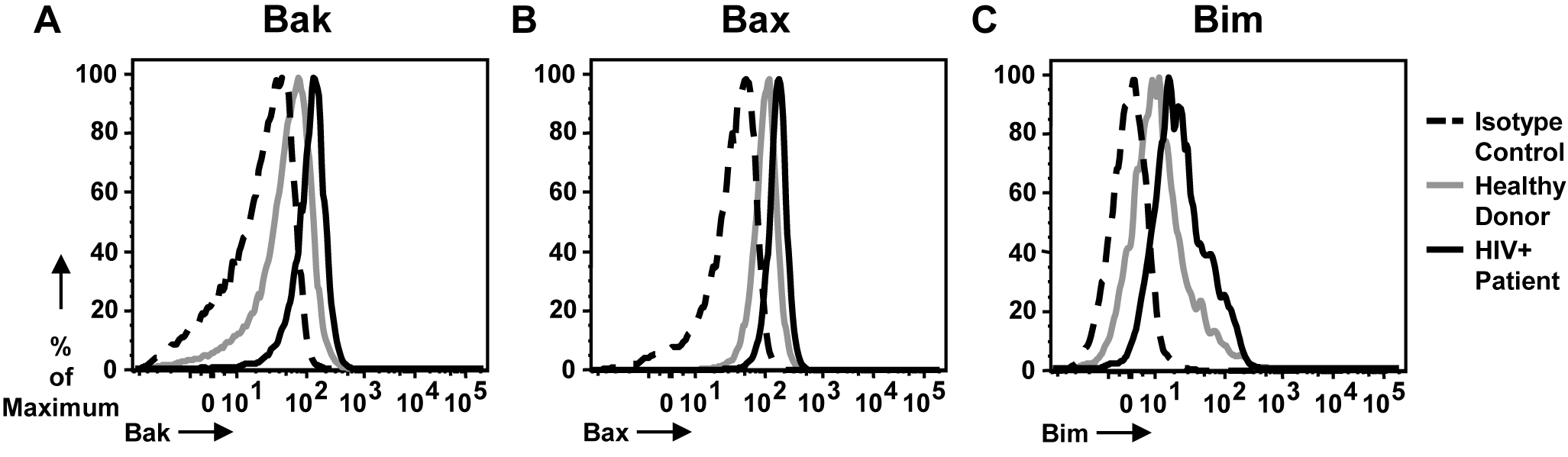

Supplement: Figure S1 — Total levels of Bak, Bax and Bim are elevated in CD4+ T cells from HIV-1-infected patients, relative to healthy donors. Representative primary data depicting mean fluorescence intensity (MFI) of total (A) Bak, (B) Bax and (C) Bim from ex vivo CD4+ T cells in an HIV-1-infected donor, as compared to healthy subject. (TIF) [file ppat.1003658.s001.tif]

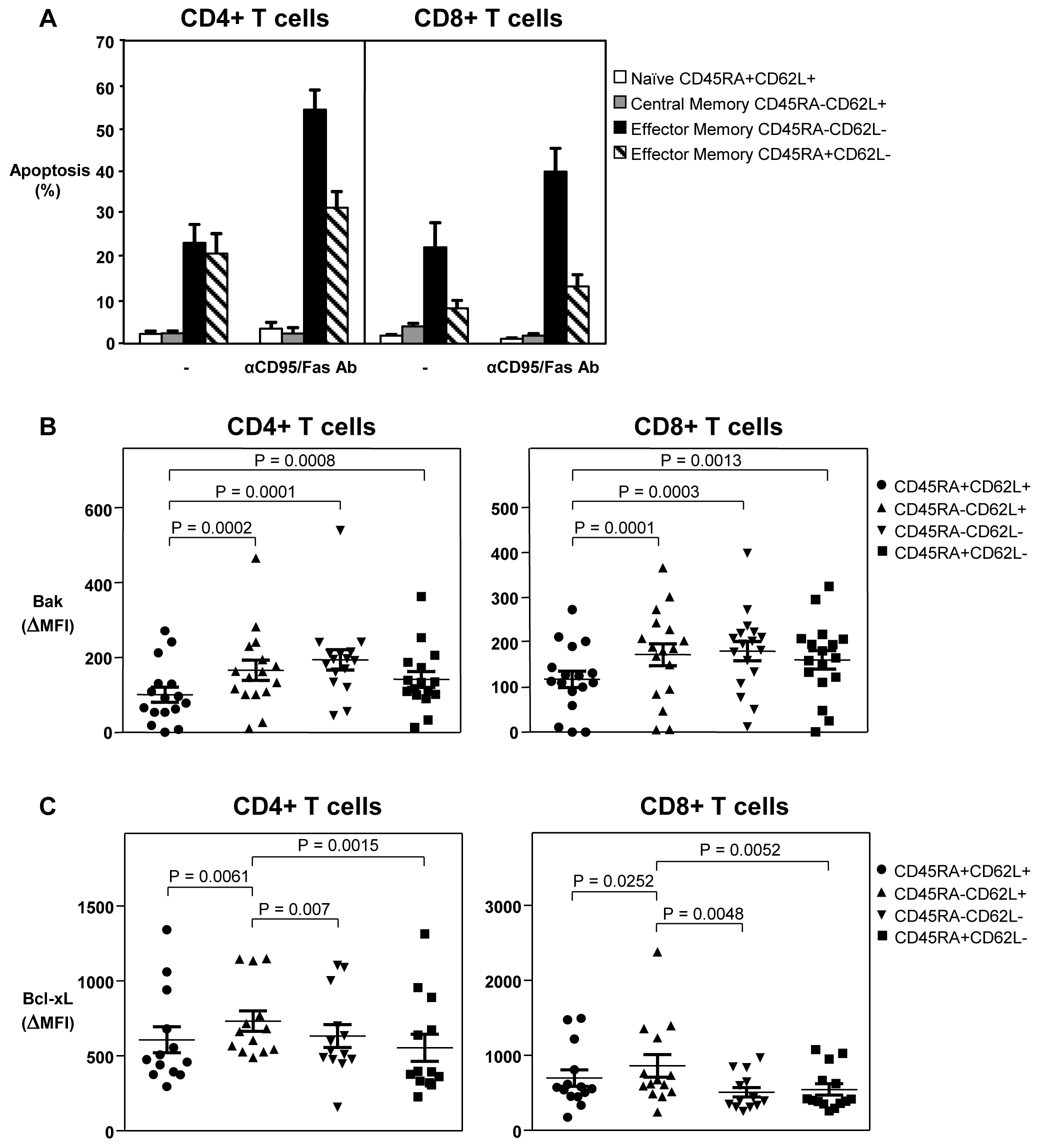

Supplement: Figure S2 — Apoptosis sensitivity of T cells in HIV-1 disease depends on the cellular differentiation state and the balance of pro- and anti-apoptotic molecules. (A) Spontaneous and CD95/Fas-mediated apoptosis of memory subpopulations of CD4+ T cells and CD8+ T cells from HIV-1-infected individuals. Levels of total (B) Bak (n = 17) and (C) Bcl-xL (n = 14) in memory subpopulations of CD4+ T cells and CD8+ T cells from HIV-1-infected donors. Each data point represents an individual HIV-1-infected patient. P values were calculated by using the Student's t-test for paired samples. (TIF) [file ppat.1003658.s002.tif]

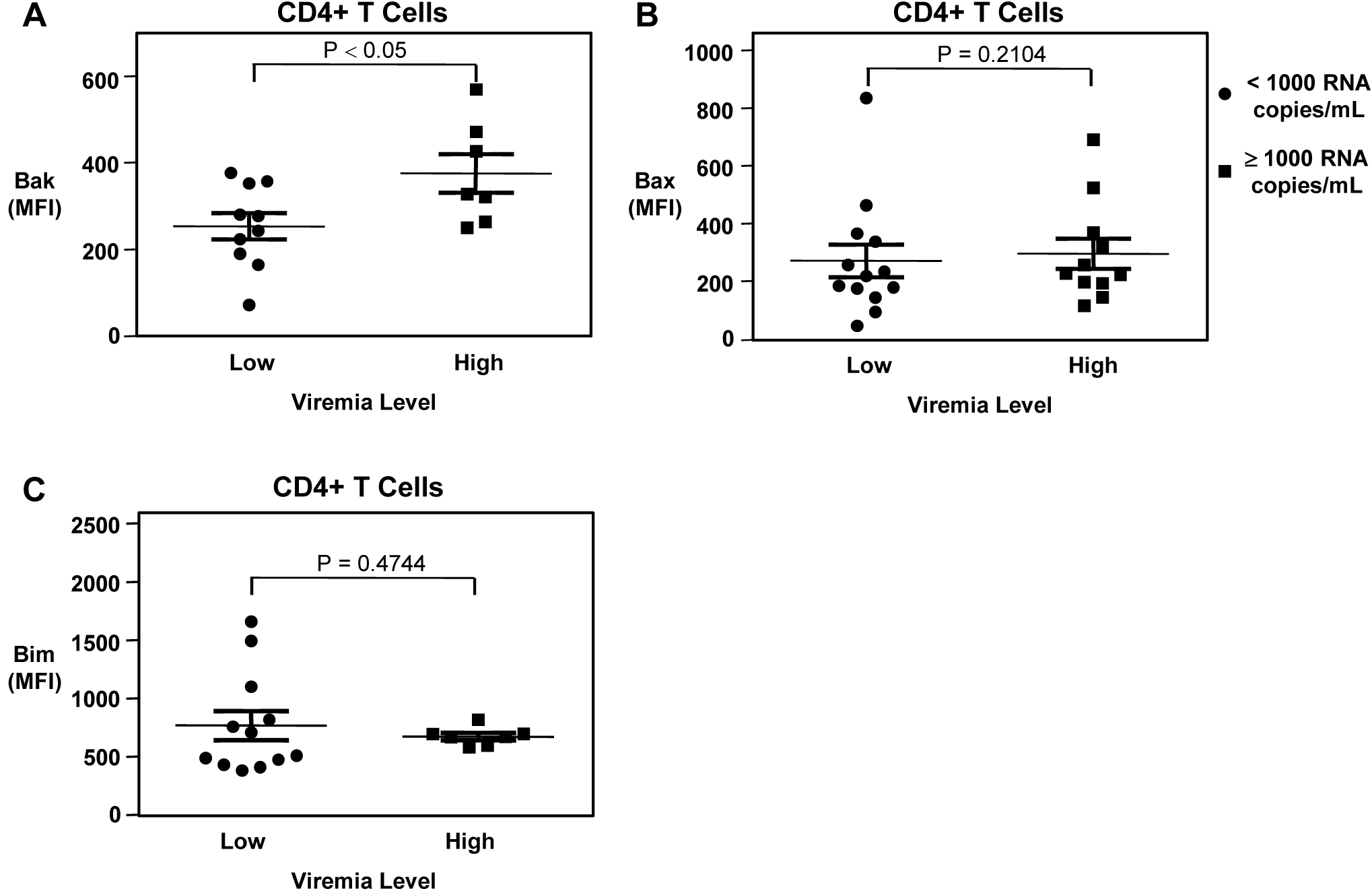

Supplement: Figure S3 — Bak levels are increased in HIV-1-infected donors with high viral loads, relative to patients with low viral loads. Levels of total (A) Bak (low: n = 10; high: n = 7), (B) Bax (low: n = 13; high: n = 11) and (C) Bim (low: n = 12; high: n = 7) in HIV-1-infected individuals with low-level (<1000 HIV RNA copies/ml) and high-level viremia (≥1000 HIV RNA copies/ml). P values for pro-apoptotic molecules were calculated by using the Student's t-test for unpaired samples. (TIF) [file ppat.1003658.s003.tif]

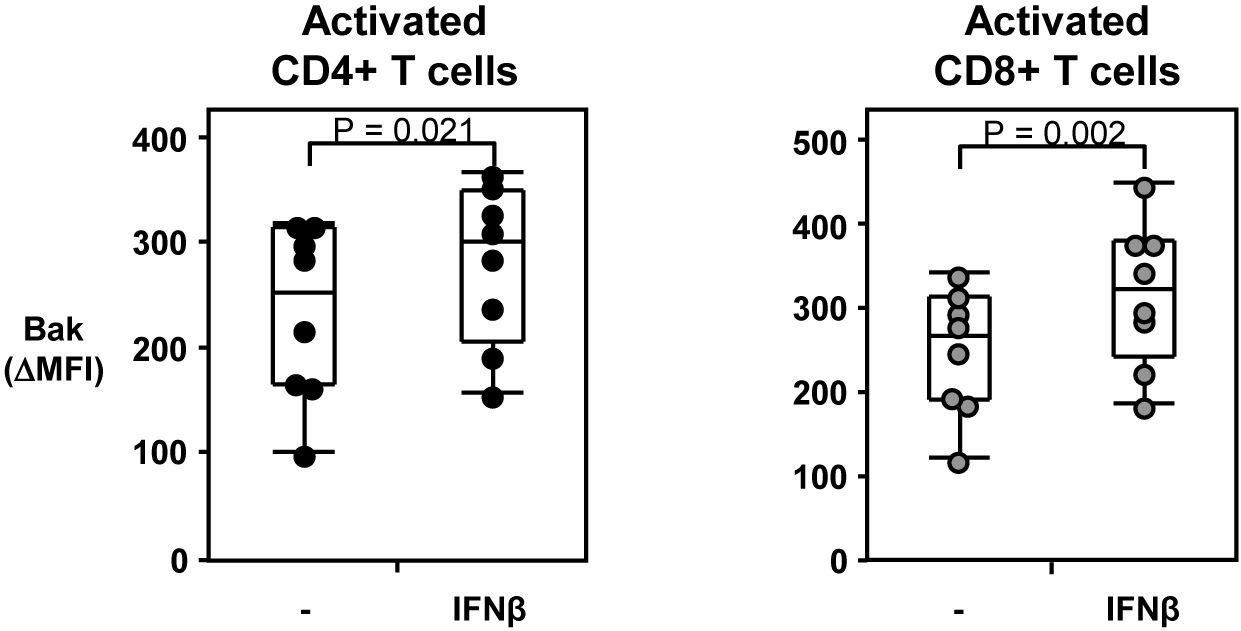

Supplement: Figure S4 — IFNβ upregulates Bak expression in activated T cells from healthy donors. Bak expression shown in CD4+ T cells and CD8+ T cells from healthy donors after PBMC were activated with plate-bound anti-CD3 antibody and untreated or treated with IFNβ (1000 U/ml) for 72 hours. Each filled circle represents one healthy donor (n = 8). Lines indicate 10% and 90% and the boxes depict 25%, median and 75% quantiles. P values were calculated by using the Student's t- test for paired samples. (TIF) [file ppat.1003658.s004.tif]

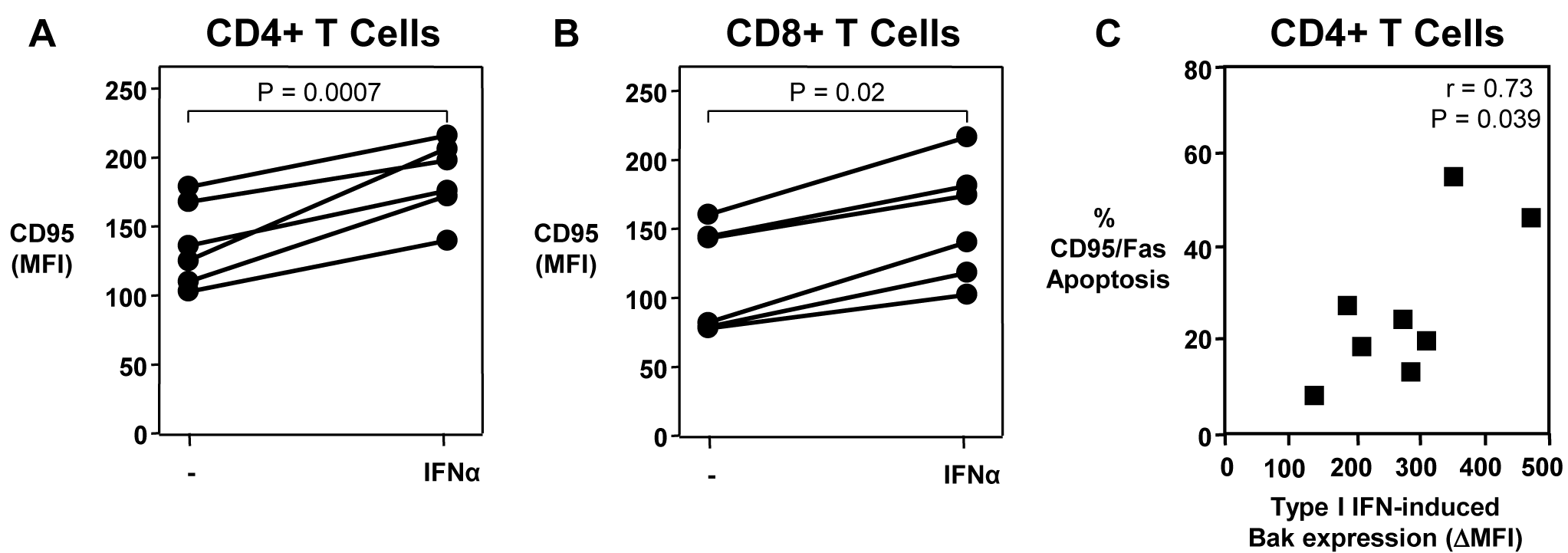

Supplement: Figure S5 — Type I IFN increases CD95 expression on healthy donor T cells and induces Bak upregulation that is directly correlated with CD95/Fas apoptosis sensitivity. CD95 expression shown on healthy donor (A) CD4+ T cells and (B) CD8+ T cells after PBMC were untreated or treated with IFNα (1000 U/ml) for 72 hours. Each filled circle represents one donor (n = 6). P values were calculated by using the Student's t-test for paired samples. (C) Pearson's correlation shown between CD4+ T cell Bak expression and CD95/Fas apoptosis sensitivity of activated CD4+ T cells following a 72 hour treatment of healthy donor PBMC with IFNβ (1000 U/ml). (TIF) [file ppat.1003658.s005.tif]

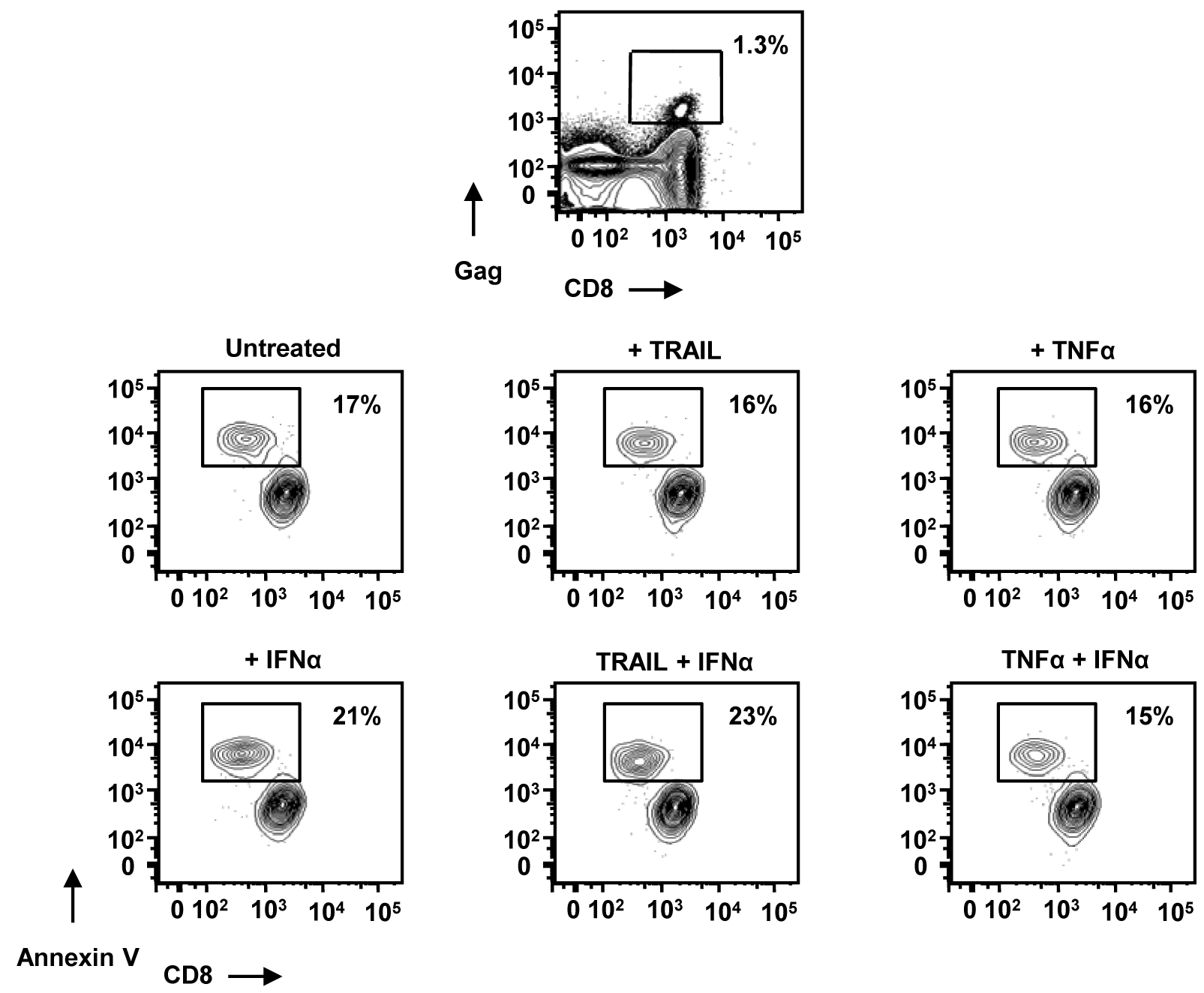

Supplement: Figure S6 — Type I IFN does not sensitize HIV-specific CD8+ T cells to TRAIL or TNFα-mediated apoptosis. PBMC were untreated or treated with IFNα (1000 U/ml) for 72 hours. Cells were then unstimulated or cultured with TRAIL (10 ng/ml) or TNFα (10 ng/ml) for 14 hours. Flow cytometric measurements of Annexin V expression were performed on Gag tetramer positive CD8+ T cells. Flow cytometry plots for one representative chronically HIV-1-infected subject are shown. (TIF) [file ppat.1003658.s006.tif]

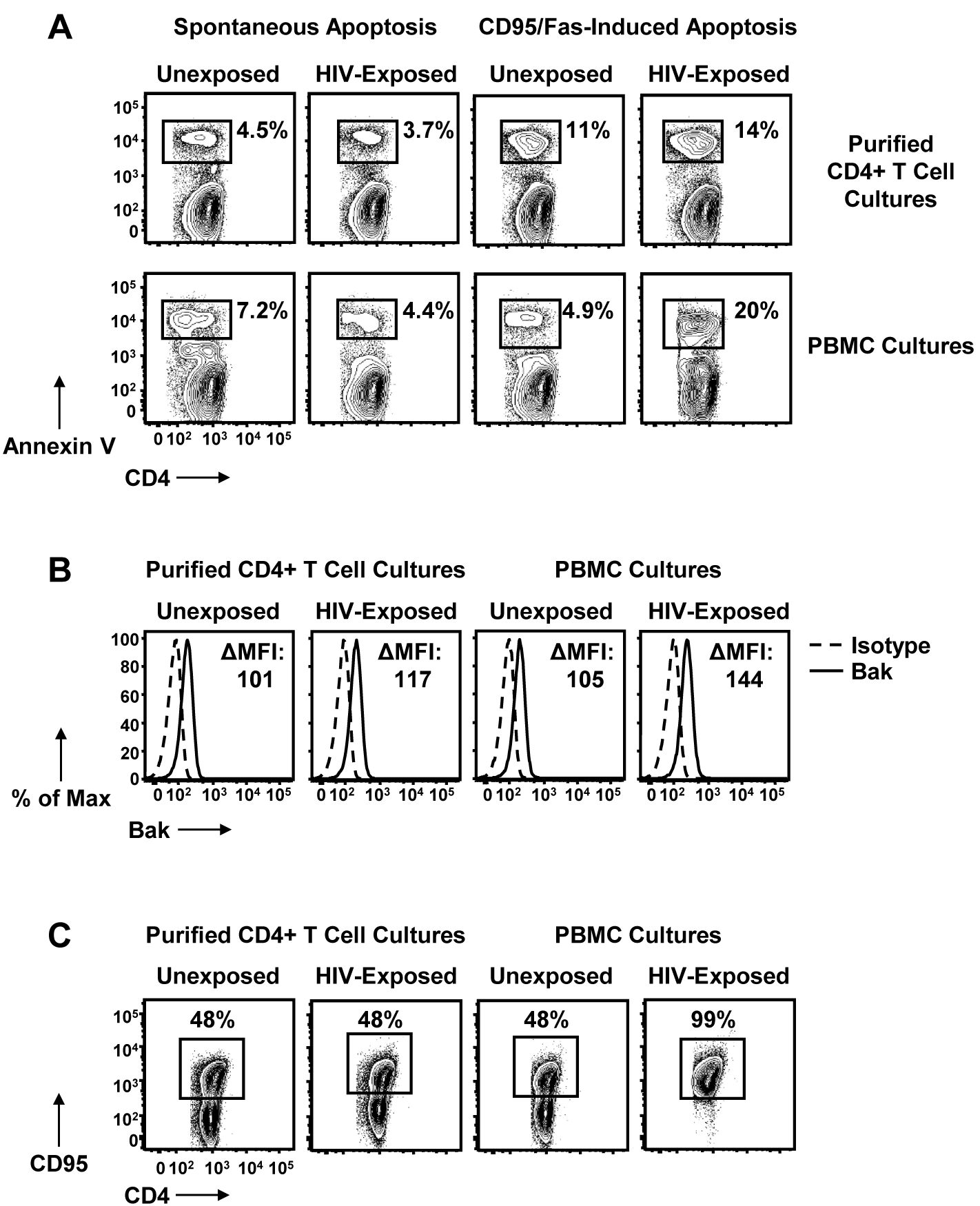

Supplement: Figure S7 — HIV-1 exposure differentially affects Fas apoptosis sensitivity, Bak expression and the frequency of CD95+ CD4+ T cells in PBMC versus purified CD4+ T cells. (A) Representative flow cytometry plots depicting spontaneous death and CD95/Fas-mediated apoptosis of purified CD4+ T cells or CD4+ T cells present in PBMC cultures from the same donor. Cells were exposed to HIV-1Ba-L for 72 hours and were subsequently left unstimulated or stimulated with solid-phase anti-CD95/Fas antibodies for 14 hours (B) Bak expression and (C) frequency of CD95-expressing CD4+ T cells in purified CD4+ T lymphocyte and PBMC cultures from one donor that were unexposed or exposed to 7×10^4^ TCID50/ml of HIV-1Ba-L for 72 hours. Results are representative of 2 independent experiments performed with 2 different healthy donors. (TIF) [file ppat.1003658.s007.tif]
